# Alveolar macrophages maintain tissue localization and gain enhanced anti-tumor activity in Lewis lung carcinoma-reprogrammed lung microenvironment

**DOI:** 10.3389/fimmu.2025.1616514

**Published:** 2025-07-24

**Authors:** Mengfei Ren, Jiaxiang Dou, Qian Yue, Liqin Ma, Hang Yu, Shengwen Shang, Shijie Wang, Jian Wang, Tingting Li, Fengqi Li

**Affiliations:** ^1^ School of Basic Medical Sciences, Center for Big Data and Population Health of IHM, Anhui Medical University, Hefei, China; ^2^ Institute of Health and Medicine, Hefei Comprehensive National Science Center, Hefei, China; ^3^ Center for Xin’an Medicine and Modernization of Traditional Chinese of IHM, Anhui University of Chinese Medicine, Hefei, China; ^4^ School of Biomedical Sciences and Engineering, South China University of Technology, Guangzhou, China; ^5^ Department of Neurology, The First Affiliated Hospital of USTC, Division of Life Sciences and Medicine, University of Science and Technology of China, Hefei, China; ^6^ State Key Laboratory of Immune Response and Immunotherapy, Division of Life Sciences and Medicine, University of Science and Technology of China, Hefei, China; ^7^ Shanxi Province Cancer Hospital, Shanxi Hospital Affiliated to Cancer Hospital, Chinese Academy of Medical Sciences, Cancer Hospital Affiliated to Shanxi Medical University, Taiyuan, China; ^8^ Department of Clinical Laboratory, First Affiliated Hospital of Anhui Medical University, Hefei, Anhui, China

**Keywords:** tumor microenvironment, alveolar macrophages, lung inflammation, Lewis lung carcinoma, extra-tumoral lung microenvironment

## Abstract

The role of alveolar macrophages (AMs) in lung carcinogenesis has been extensively studied, yielding significant insights. However, the status of AMs in tumor-bearing lungs remains incompletely characterized. Using orthotopic Lewis Lung Carcinoma (LLC) mouse models, we found that tumors induced an inflammatory extra-tumoral lung microenvironment (ETLME), distinct from the immunosuppressive tumor microenvironment (TME). T cells with an exhaustion phenotype and tumor-associated macrophages (TAMs) mainly accumulated in the TME rather than the ETLME. Surprisingly, AMs were absent from the tumor lesions and remained in the lung tissues, but they displayed a more active dynamic balance between proliferation and death in ETLME. Furthermore, AMs presented an activated phenotype characterized by upregulation of CD11b and downregulation of Siglec-F, elevated expression of inflammatory genes, and enhanced phagocytic and efferocytotic activity. Notably, AMs in ETLME retained their lipid metabolism capacity and responsiveness to external stimuli. More importantly, LLC-experienced AMs display enhanced anti-tumor ability. These findings indicate that AMs maintain their tissue localization and functional integrity within the ETLME.

## Introduction

Lung cancer is one of the most prevalent cancers worldwide, and is characterized by high incidence and mortality rates, posing a serious threat to human health and life safety (1, 2). Lung adenocarcinoma is a major subtype of non-small cell lung cancer (NSCLC) and is the most common type of lung cancer (3). The unique structure and physiological functions of the lungs create a complex immune microenvironment that facilitates tumor growth and metastasis in the lungs (4). Conversely, the tumor-reprogrammed lung immune microenvironment also profoundly influences the features of tissue-resident immune cells within the lungs (5–7). Therefore, understanding the distribution, phenotype, and function of immune cells in the tumor-reprogrammed lung microenvironment is critical for discovering new therapeutic targets and developing effective treatments.

Alveolar macrophages (AMs) are lung-resident macrophages that exist in the alveoli. They exert crucial roles in defending against inhaled pathogens, modulating immune responses, and maintaining pulmonary immune homeostasis (8, 9). AMs play roles in engulfing and catabolizing alveolar surfactants, removing debris through phagocytosis, and clearing apoptotic cells through efferocytosis (10). In addition to traditional defense and clearance functions, emerging evidence suggests that AMs also significantly influence lung cancer initiation and progression. During tumor formation, AMs have been observed to accumulate close to lung tumor cells and promote tumor growth (11). In EGFR-driven lung adenocarcinomas, AMs support tumor growth by providing nutrients (12). Moreover, AMs also contribute to the formation of the premetastatic lung niche for breast cancer (13, 14). Depletion of AMs has been shown to reduce tumor invasiveness or growth in EGFR-mutant lung adenocarcinoma (15), Kras^G12D^ and p53-deficient lung cancer (11), and chemically induced lung adenocarcinoma (16) mouse models. While AMs have been shown to play opposite roles in breast cancer in another study, they interact with disseminated breast cancer cells in the alveolar space and suppress metastasis *via* the TGF-β2/TGF-βRIII signaling pathway (17). However, previous studies on the function of AMs in tumors have mainly focused on the early stages of tumorigenesis (11–18), with few studies investigating their status after tumor formation.

During tumor progression, cancer cells employ multiple strategies to evade immune surveillance, including downregulating antigen presentation and inducing inhibitory immune checkpoint molecules (19, 20). Additionally, they co-opt immune cells, such as neutrophil subsets (21), macrophages (12), and regulatory T cells (22), to shape an immunosuppressive tumor microenvironment (TME), fostering immune escape, vascular remodeling, and treatment resistance. Previous studies have shown that tumor-associated macrophages (TAMs) in lung tumor lesions consist of two distinguishable macrophage populations, tissue-resident macrophages (mainly AMs) and monocyte-derived macrophages, particularly in the early stage of tumor growth (11). Investigating the role of AMs within this tumor-reprogrammed microenvironment following tumor formation may provide new insights for lung cancer therapy.

For this purpose, we utilized the Lewis lung cancer (LLC) mouse model, a well-known mouse model of lung cancer that recapitulates many of the features of human lung adenocarcinoma. We found that LLC created an inflammatory lung microenvironment with significant changes in the composition of immune cells throughout the lung, but the number of AMs did not change. Interestingly, a comparison of immune cell composition between dissected tumor nodules and surrounding lung tissue revealed that T cells with an exhaustion phenotype were predominantly localized to tumor lesions, while adjacent lung tissues showed minimal presence. Additionally, AMs are rarely found in tumor lesions. AMs exhibited an activated phenotype characterized by upregulated inflammatory gene expression, increased phagocytosis and efferocytosis functions, and a more active dynamic equilibrium between proliferation and death in LLC-bearing lungs. Importantly, AMs in LLC-bearing lungs retained normal lipid metabolism, the capacity to respond to external stimuli, and gained enhanced anti-tumor activity. Our findings demonstrate that AMs maintain their normal distribution and functional integrity in the LLC-reprogrammed lung microenvironment, indicating their potential as therapeutic targets in lung cancer.

## Results

### LLC creates an inflammatory microenvironment in the lungs

**Figure 1:**
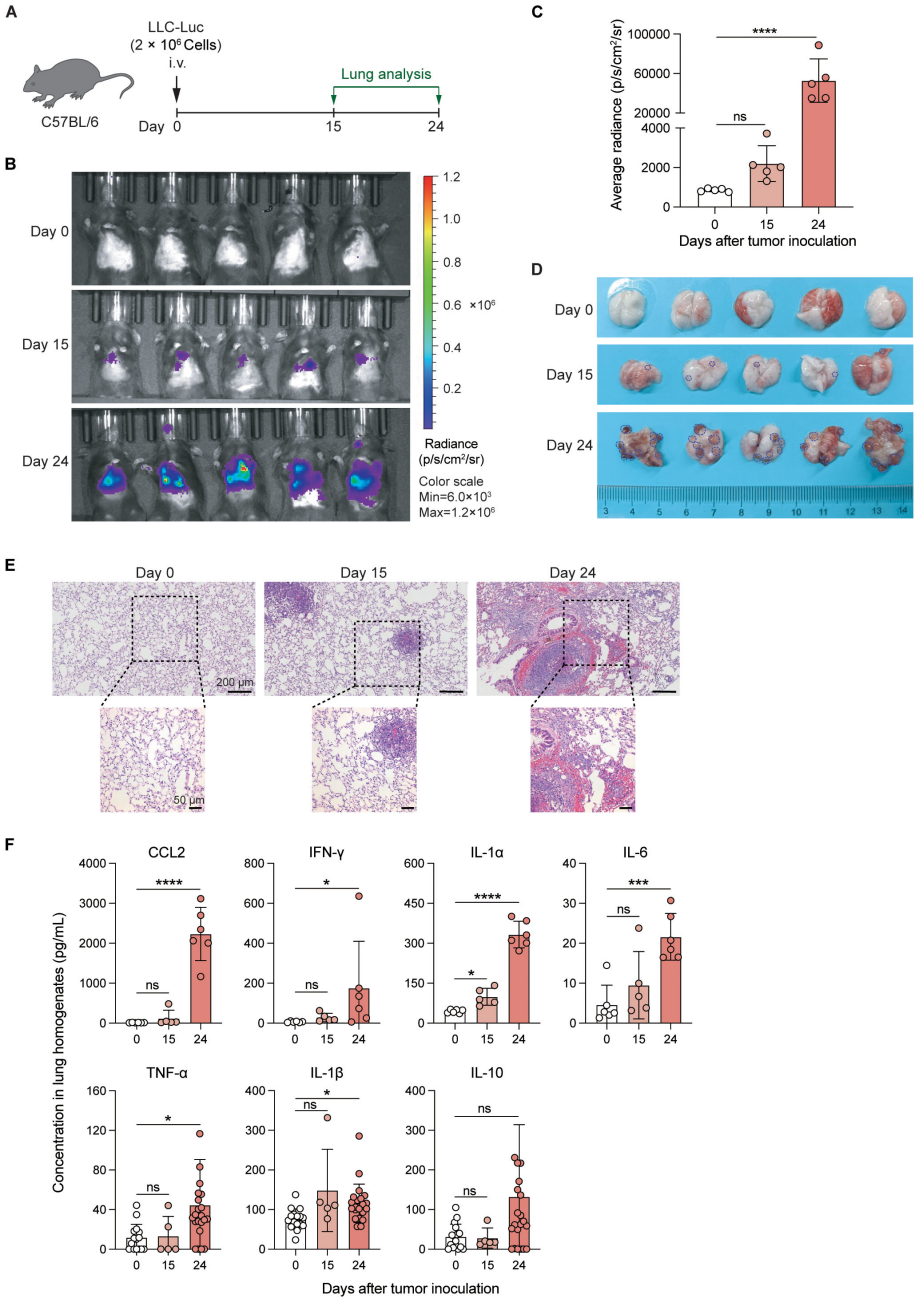
LLC creates an inflammatory microenvironment in the lungs. **(A)** Schematic of the experimental design. Luciferase-expressing Lewis lung carcinoma cells (LLC-Luc) were intravenously (i.v.) inoculated into C57BL/6 mice, and tumor progression was analyzed on days 0, 15, and 24. **(B, C)** Luciferin-based *in vivo* tumor imaging **(B)** and quantification of the average radiance of luciferin signals in the lung region at various time points post-inoculation **(C)**. **(D)** Macroscopic examination of lung tumor nodules; the dark blue dashed line shows the boundary of the representative tumor nodules. **(E)** Representative histopathological images of lungs at different stages of tumor progression. **(F)** Concentrations of proinflammatory chemokines/cytokines, including CCL2, IFN-γ, IL-1α, IL-6, TNF-α, IL-1β, and IL-10 in lung tissue homogenates during tumor progression. The data are presented as mean ± SD (n = 5–6 mice per group in C and upper panel of F, n = 5–22 mice per group in lower panel of F). One-way ANOVA was used for **(C, F)**, with comparisons made to day 0. “ns”, not significant; *P < 0.05, ***P < 0.001, and ****P < 0.0001.

To characterize the changes in the lung microenvironment during orthotopic tumor progression, we utilized the LLC lung tumor model, a well-established mouse model of lung cancer that recapitulates many features of human lung adenocarcinoma. C57BL/6 mice were intravenously (i.v.) injected with luciferase-expressing LLC cells (LLC-Luc) (**Figure 1A**). Tumors were observed in the lungs at 15 days post-inoculation *via* luciferin-based *in vivo* imaging (**Figures 1B, C**), and small tumor nodules were occasionally visible in the lungs at this time (**Figure 1D**). At 24 days post-inoculation, luciferin signals were intensified in the lungs (**Figures 1B, C**), and multiple large tumor nodules were observed across all the lung lobes (**Figure 1D**). Histopathological analysis of the lungs further supported the above findings (**Figure 1E**). In addition, compared with slight inflammatory cell infiltration at 15 days post-inoculation, extensive infiltration of inflammatory cells was observed in the lungs at 24 days post-inoculation (**Figure 1E**). At 15 days post-inoculation, only IL-1α, among the detected chemokines and cytokines, slightly increased in the lung homogenates. Several inflammatory chemokines and cytokines, including C-C motif chemokine ligand 2 (CCL2), interferon-gamma (IFN-γ), interleukin-1 alpha (IL-1α), interleukin-6 (IL-6), tumor necrosis factor-alpha (TNF-α), and interleukin-1 beta (IL-1β), were significantly elevated at 24 days post-inoculation (**Figure 1F**). The CCL2 is critical for the recruitment of inflammatory immune cells into the lung, and IFN-γ, IL-1α, IL-6, TNF-α, and IL-1β are important inflammatory mediators that activate macrophages and stimulate adaptive immune responses. While the levels of immunosuppressive cytokine, IL-10, did not display significant changes (**Figure 1F**). Therefore, the increased inflammation in the lungs with the progression of LLC indicates the breakdown of lung immune homeostasis (18), which might affect the resident immune cells in the lungs, such as AMs.

### LLC significantly alters the composition of lung immune cells

**Figure 2:**
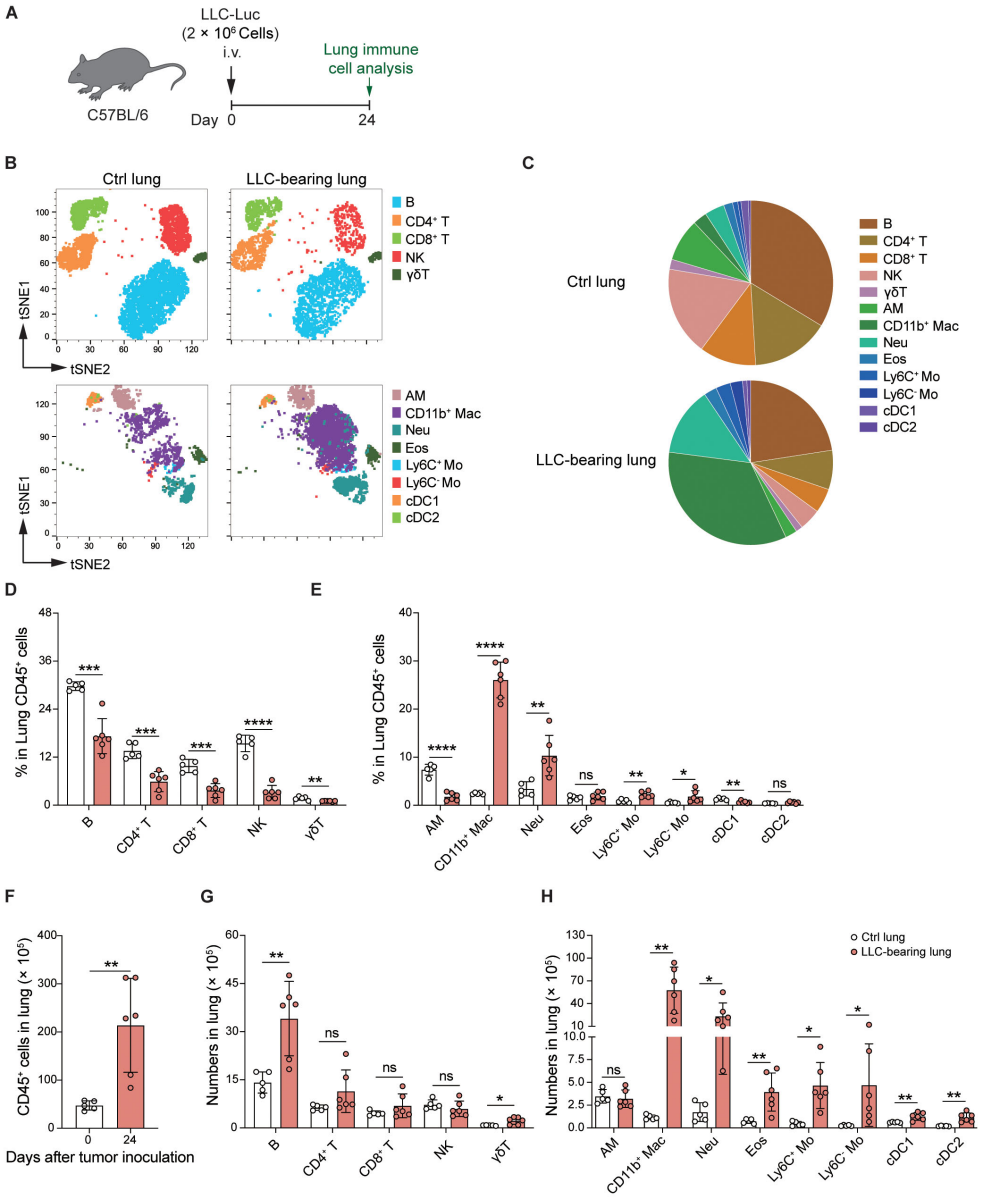
LLC significantly alters the composition of lung immune cells. **(A)** Schematic of the experimental design. LLC-Luc were i.v. inoculated into C57BL/6 mice. Lung immune cells from LLC-bearing mice at 24 days post-inoculation and control mice were analyzed. **(B)** t-distributed Stochastic Neighbor Embedding (t-SNE) plots depicting the clustering and distribution of immune cell types in the lungs. The cell types are color-coded as indicated in the legend. Lymphocyte subsets include B cells, CD4^+^ T cells, CD8^+^ T cells, and γδ T cells. The myeloid cell subsets include alveolar macrophages (AM), CD11b^+^ macrophages (CD11b^+^ Mac), neutrophils (Neu), eosinophils (Eos), Ly6C^+^ and Ly6C^-^ monocytes (Mo), conventional dendritic cells 1 (cDC1), and cDC2. **(C)** Pie charts showing the composition of different immune cell populations in the lungs. **(D, E)** Percentages of lymphocyte **(D)** and myeloid cell subsets **(E)** among CD45^+^ lung cells. **(F)** Total numbers of CD45^+^ cells in the lungs. **(G, H)** Absolute numbers of lymphocyte **(G)** and myeloid cell subsets **(H)** among CD45^+^ lung cells. The data are presented as means ± SDs (n = 5–6 mice per group). Unpaired *t*-tests were used in **(D-H)**, “ns” indicates not significant, *P < 0.05, **P < 0.01, ***P < 0.001, and ****P < 0.0001.

To characterize the LLC-created inflammatory microenvironment in the lungs comprehensively, we first analyzed whole lung immune cells from control mice and LLC-bearing mice at 24 days post-inoculation (**Figure 2A**). The gating strategy of different immune cell populations is shown in **Supplementary Figure S1**. AMs were identified as CD45^+^Ly6G^-^CD64+Siglec-F^hi^CD11b^-^ cells (**Supplementary Figure S1**). The t-SNE plots and pie charts based on the data of flow cytometry analysis revealed that the proportions of the various immune cell subsets were significantly altered (**Figures 2B, C**). Among them, the proportions of lymphocyte subsets, including B, CD4^+^ T, CD8^+^ T, NK, and γδT cells, were decreased in the LLC-bearing lungs (**Figure 2D**), and except for AMs, the proportions of myeloid cell subsets, such as CD11b^+^ macrophages (Mac), neutrophils (Neu), and monocytes (Mo) were increased (**Figure 2E**). Counting the absolute number of cells revealed that the total number of CD45^+^ cells was significantly increased in the LLC-bearing lungs (**Figure 2F**), which was mainly attributable to the increase in B cells, γδT cells and most myeloid cell subsets, but the numbers of CD4^+^ T, CD8^+^ T, NK cells and AMs did not increase (**Figures 2G, H**). Thus, LLC tumor growth promotes the infiltration of several myeloid cell subsets in the lungs, but the number of AMs remains stable.

Next, we analyzed T-cell subsets in control and tumor-bearing lungs *via* flow cytometry (**Supplementary Figure S2A**). CD62L and CD44 were stained to distinguish naïve T cells, central memory T cells (Tcm), and effector T/effector memory T cells (Tem/Teff) (23, 24). We found that the proportion of naïve T cells (CD62L^+^CD44^-^) remained unchanged on day 15 but decreased by day 24 post-tumor inoculation. In contrast, the percentage of Tcm (CD62L^+^CD44^+^) increased on day 15 before declining on day 24, and the percentage of Tem/Teff (CD62L^-^CD44^+^) increased on day 15 and day 24 (**Supplementary Figures S2B-E**), indicating that LLC alters the composition of T-cell subsets in the lungs. Analysis of the expression of the inhibitory receptors PD-1 and Tim-3 (25, 26) on CD4^+^CD44^+^/CD8^+^CD44^+^ T cells revealed elevated frequencies of the PD-1^+^Tim3^+^ subsets by day 24 (**Supplementary Figures S2F, G**), revealing that T cells display an exhaustion phenotype at the late stage of lung tumor growth. These findings demonstrate that LLC tumor growth significantly alters the composition of lung immune cells.

### AMs are predominantly localized in tissues and rarely infiltrate tumor lesions

**Figure 3:**
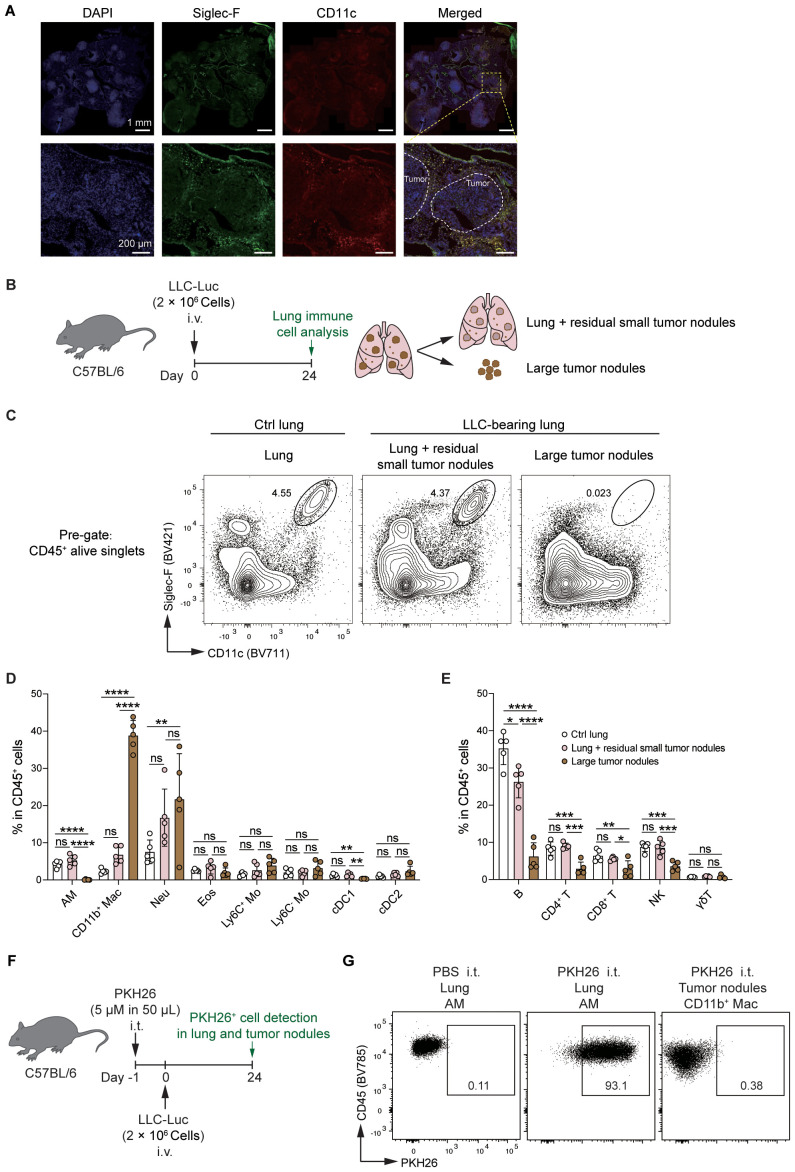
AMs remain resident in the lungs but do not infiltrate tumor lesions. **(A)** Representative immunofluorescence images of tumor-bearing lungs. Nucleus were stained with DAPI, Siglec-F^+^CD11c^+^ cells were defined as AMs. White circles indicate the tumor lesions. **(B)** Schematic of the experimental design for FACS analysis. LLC-Luc cells were i.v. inoculated into C57BL/6 mice. On day 24 post-inoculation, large tumor nodules were dissected from the lung tissues, and the tumor-bearing lungs were separated into two fractions: (1) large tumor nodules and (2) lungs containing residual small tumor nodules. The immune cell composition was then analyzed for both fractions. **(C)** Representative flow cytometry dot plots of AMs (CD11c^hi^Siglec-F^hi^). The numbers indicate the percentage of AMs among live CD45^+^ singlets. **(D, E)** Percentages of myeloid cells **(D)** and lymphocyte subsets **(E)** among CD45^+^ cells. **(F)** Schematic of the experimental design for PKH26 labeling. LLC-Luc cells were i.v. inoculated into C57BL/6 mice 1 day after i.t. treatment with 5μM of PKH26 in 50 μL. On day 24 post-inoculation, PKH26-labelled AMs were detected in lung tissue and tumor nodules. **(G)** Representative flow cytometry dot plots of PKH26-labelled AMs, PBS i.t. treated mice as control. The data in D and E are presented as means ± SDs (n = 5 mice per group). One-way ANOVA was used in **(C, D)**, “ns” indicates not significant, *P < 0.05, **P < 0.01, ***P < 0.001, and ****P < 0.0001.

To determine the influence of the tumor microenvironment (TME) and extra-tumoral lung microenvironment (ETLME) on AMs, we first detected the localization of AMs in LLC-bearing lungs. By using immunofluorescence staining for tumor-bearing lung sections, we observed that AMs were distributed closely to the border of the tumor nodules but did not infiltrate the tumor lesions (**Figure 3A**), implying that AMs might retain their tissue-resident properties and are not recruited into tumor lesions. To further confirm the distribution of AMs in tumor-bearing lungs, we dissected large tumor nodules from lung tissues, and then immune cells were isolated from large tumor nodules (representing the TME) and the remaining lung tissues containing residual small tumor nodules (representing the ETLME) (**Figure 3B**). AMs were detected only in the remaining lung tissues but not in large tumor nodules (**Figures 3C, D**), confirming that AMs maintain their tissue localization and are not recruited into tumor lesions. In addition, the significantly increased percentage (**Figure 2E**) and number (**Figure 2H**) of CD11b^+^ macrophages in LLC-bearing lungs were predominantly present in tumor nodules and were rare in the remaining lung tissues (**Figure 3D**). These increased CD11b^+^ macrophages might be TAMs, which have been proven to be the key drivers of T-cell exhaustion in tumor environments (27, 28). Consistently, the proportion of different lymphocyte subsets was reduced in the tumor nodules (**Figure 3E**). Naïve T and Tcm subsets were reduced in large tumor nodules but unchanged in lung tissues. In contrast, Tem/Teff subsets were elevated in tumor nodules but not in adjacent lung tissue (**Supplementary Figures S3A-C**). PD-1^+^Tim3^+^ cells were increased in CD4^+^CD44^+^/CD8^+^CD44^+^T cells, indicating that the CD4^+^ and CD8^+^ T cells with exhaustion phenotype were predominantly present in tumor nodules (**Supplementary Figure S3D**). Next, to further explore the possibility that AMs lose their typical surface markers and differentiate into TAMs in TME, AMs were labelled 1 day before tumor inoculation with PKH26, a fluorescent dye that could specifically label AM by intratracheal administration for longer than two months (9). We found that PKH26-labelled cells rarely appeared in tumor nodules (**Figures 3F, G**), which further confirmed that the tissue distribution of AMs and excluded the possibility that AMs differentiated into TAMs. Thus, there are significant differences in the composition of immune cells in TME and ETLME in LLC-bearing mice, and AMs are predominantly localized in tissues and rarely infiltrate tumor lesions.

### AMs are dynamically stabilized in the LLC-reprogrammed ETLME

**Figure 4:**
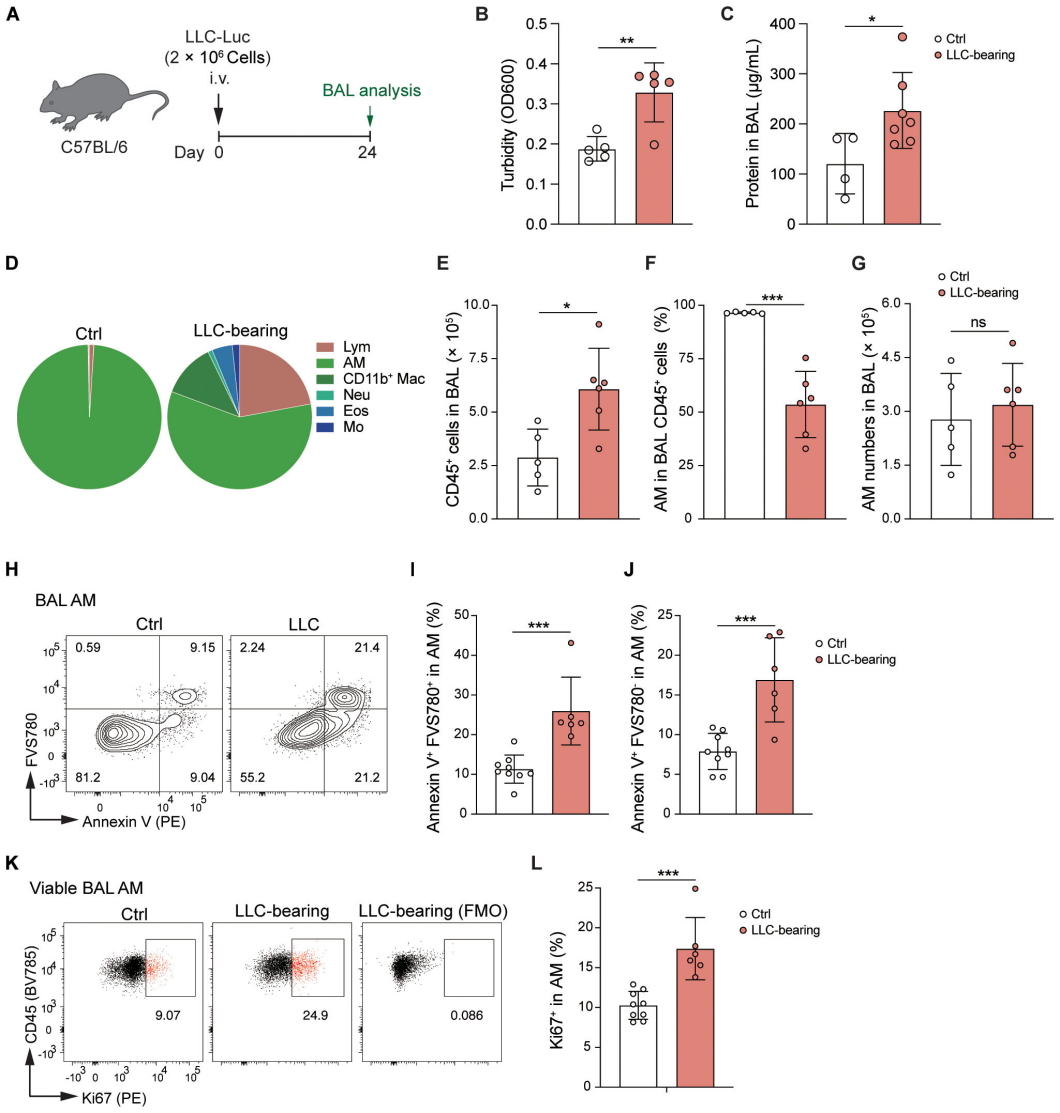
AMs are dynamically stabilized in the LLC-reprogrammed ETLME. **(A)** Schematic of the experimental design. LLC-Luc were i.v. inoculated into C57BL/6 mice. Bronchoalveolar lavage (BAL) fluid was analyzed 24 days post-inoculation. **(B, C)** BAL fluid analysis showing turbidity (OD600) **(B)** and total protein concentration **(C)**. **(D)** Pie charts showing the composition of different immune cell subsets in BAL: lymphocytes (Lym), AM, CD11b^+^ Mac, Neu, Eos, and Mo. **(E)** Total numbers of CD45^+^ cells in BAL fluid. **(F)** Percentages of AM in BAL CD45^+^ cells. **(G)** Absolute numbers of AMs in BAL fluid. **(H)** Flow cytometric analysis of Annexin V and FVS780 in BAL AM. **(I, J)** Percentages of Annexin V^+^FVS780^-^ (the early-stage apoptotic) cells **(I)** and Annexin V^+^FVS780^+^ (late-stage apoptotic and necrotic) cells **(J)** in BAL AM. **(K)** Flow cytometric analysis of Ki67 expression in BAL AM, and with a fluorescence minus one (FMO) control to assess cell proliferation. **(L)** Percentages of Ki67^+^ cells in BAL AM. The data are presented as means ± SDs (n = 4–9 mice per group). Unpaired *t*-tests were used, “ns” indicates not significant, *P < 0.05, **P < 0.01, and ***P < 0.001.

Next, we further analyzed the status of AMs in LLC-bearing mice (**Figure 4A**). Bronchoalveolar lavage (BAL) fluid was harvested, and the turbidity and protein levels in the BAL fluid of LLC-bearing mice were significantly increased (**Figures 4B, C**), indicating that LLC-bearing conditions changed the microenvironment of the alveoli. These findings were further supported by the analysis of immune cells in the alveoli, where there was infiltration of other immune cells and an increase in the total number of CD45^+^ cells in the alveoli of the LLC-bearing mice compared with the control mice, which had only AMs in the alveoli (**Figures 4D, E**). However, consistent with the count of AMs at the whole lung level, the percentage and absolute number of AMs in the BAL did not increase in LLC-bearing mice (**Figures 4F, G**). During homeostasis, mature AMs are maintained through self-renewal (29), and low levels of AM death and the degree of AM proliferation are detected in control mice (**Figures 4H-L**). However, both the degree of death and the degree of AM proliferation were increased under the influence of the LLC-reprogrammed microenvironment (**Figures 4H-L**), which might explain why the number of AMs did not change with the LLC progression. Thus, AMs are dynamically stabilized in the LLC-reprogrammed ETLME.

### AMs acquire an activated pro-inflammatory phenotype in LLC-reprogrammed ETLME

**Figure 5:**
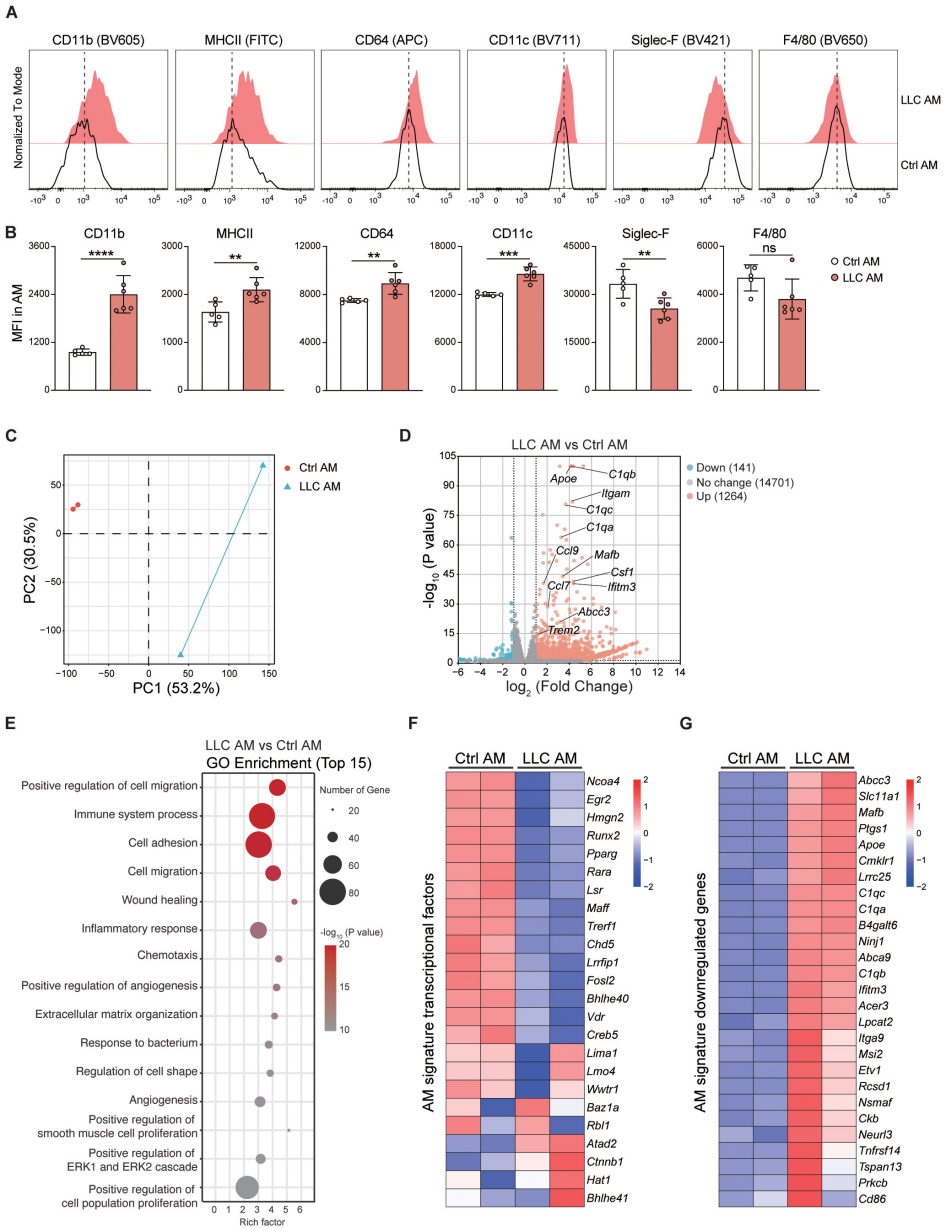
AMs acquire an activated pro-inflammatory phenotype in LLC-reprogrammed ETLME. **(A)** Representative histograms comparing surface marker expression (CD11b, MHC II, CD64, CD11c, Siglec-F, and F4/80) on control AMs (black line, Ctrl AM) and LLC-experienced AMs (red-filled, LLC AM) at 24 days post-tumor inoculation. **(B)** Mean fluorescence intensity (MFI) of CD11b, MHCII, CD64, CD11c, Siglec-F, and F4/80 in ctrl and LLC AMs. **(C)** Principal component analysis (PCA) of transcriptional profiles in LLC AM versus Ctrl AM. **(D)** Volcano plot showing differentially expressed genes (DEGs) between LLC AM and Ctrl AM, key DEGs are labelled. **(E)** Gene Ontology (GO) enrichment analysis of the top 15 biological processes associated with DEGs in LLC AM compared with Ctrl AM. The size of the points represents the number of genes, and the color represents the -log_10_ (P value). **(F)** Heatmap of AM signature transcription factor expression levels in the Ctrl AM and LLC AM. **(G)** Heatmap of AM signature downregulated genes showing their expression levels in Ctrl AM and LLC AM. The data are presented as means ± SDs in B (n = 5–6 mice per group). Unpaired *t*-tests were used, “ns” indicates not significant, **P < 0.01, ***P < 0.001, and ****P < 0.0001.

Although there was no change in the number of AMs in LLC-bearing lungs, the increase in death and proliferation levels implied a change in AM status. Indeed, AMs upregulated the expression of CD11b, MHCII, CD64, and CD11c and downregulated the expression of Siglec-F in an LLC-reprogrammed lung microenvironment (**Figures 5A, B**), indicating that AMs had adopted an activated phenotype. To further characterize AM features in the LLC-reprogrammed lung microenvironment, we isolated AMs from BAL by removing the floating cells after 30 minutes of culture. Then RNA sequencing analysis was performed to compare the gene expression patterns of AMs from control mice and LLC-bearing mice. Principal component analysis (PCA) and volcano plots based on the transcriptome analysis further revealed that the AMs of LLC-bearing mice were different from normal AMs and upregulated the expression of abundant genes (**Figures 5C, D**). Gene Ontology (GO) enrichment analysis of biological processes revealed that the upregulated expression of genes in AMs of LLC-bearing mice was associated mainly with inflammatory responses, cell adhesion, cell migration, and positive regulation of cell population proliferation (**Figure 5E**). Specifically, AMs of LLC-bearing mice downregulated the majority (13 of 24) of AM signature transcription factors (30) compared with AMs from naïve mice, including *Ncoa4*, *Hmgn2*, *Runx2*, *Pparg*, *Rara*, *Lsr*, *Maff*, *Trerf1*, *Chd5*, *Lrrfip1*, *Fosl2*, *Bhlhe40*, *Vdr* and *Creb5* (**Figure 5F**). Additionally, all 27 AM signature downregulated genes (30) were upregulated in tumor-experienced AMs compared with AMs from naïve mice (**Figure 5G**), further indicating the activation of AMs in the tumor-bearing lung microenvironment. Indeed, LLC-experienced AMs significantly increased the expression of proinflammatory genes, including multiple cytokines (e.g. *Il34*, *Il23a*, *Il6*, and *Ltb*), chemokines (e.g. *Ccl24*, *Cxcl9*, *Cxcl16*, *Ccl8*, *Cxcl3*, *Ppbp* (Cxcl7), *Pf4* (Cxcl4), *Cxcl2*, *Ccl5*, *Cxcl1*, *Ccl7*, *Ccl2*, *Ccl9*, *Cxcl10*, and *Ccl4*), cytokine receptors (e.g. *Il21r*, *Il7r*, *Il23r*, *Il18r1*, *Il18rap*, *Il1rl1*, *Il2rb*, *Osmr*, *Il12rb1*, *Il17rc*, and *Il1r2*), and chemokine receptors (e.g. *Ccr7*, *Ccr3*, *Ccr5*, *Cxcr6*, *Cx3cr1*, and *Cxcr2*) (**Supplementary Figures S4A, B**). Members of the TNF ligand superfamily, such as *Tnfsf8* (alias CD30L) and *Tnfsf14* (alias Light), and members of the TNF receptor superfamily, including *Tnfrsf9* (alias 4-1BB), *Tnfrsf14* (alias HVEM), and *Tnfrsf10b* (alias DR5), were also upregulated. In contrast, the expression of the inhibitory cytokine gene *Tgfb2* was reduced (**Supplementary Figures S4A, B**). Consistent with the protein-level observations (**Figures 5A, B**), the expression of *Itgam* (encoding CD11b) was significantly increased, and the expression of *Siglecf* was significantly decreased (**Supplementary Figure S4C**).

Collectively, these findings demonstrate that AMs acquire an activated and inflammatory phenotype in the LLC-reprogrammed ETLME.

### AMs exhibit enhanced phagocytic and efferocytotic capacity in the LLC-reprogrammed ETLME

**Figure 6:**
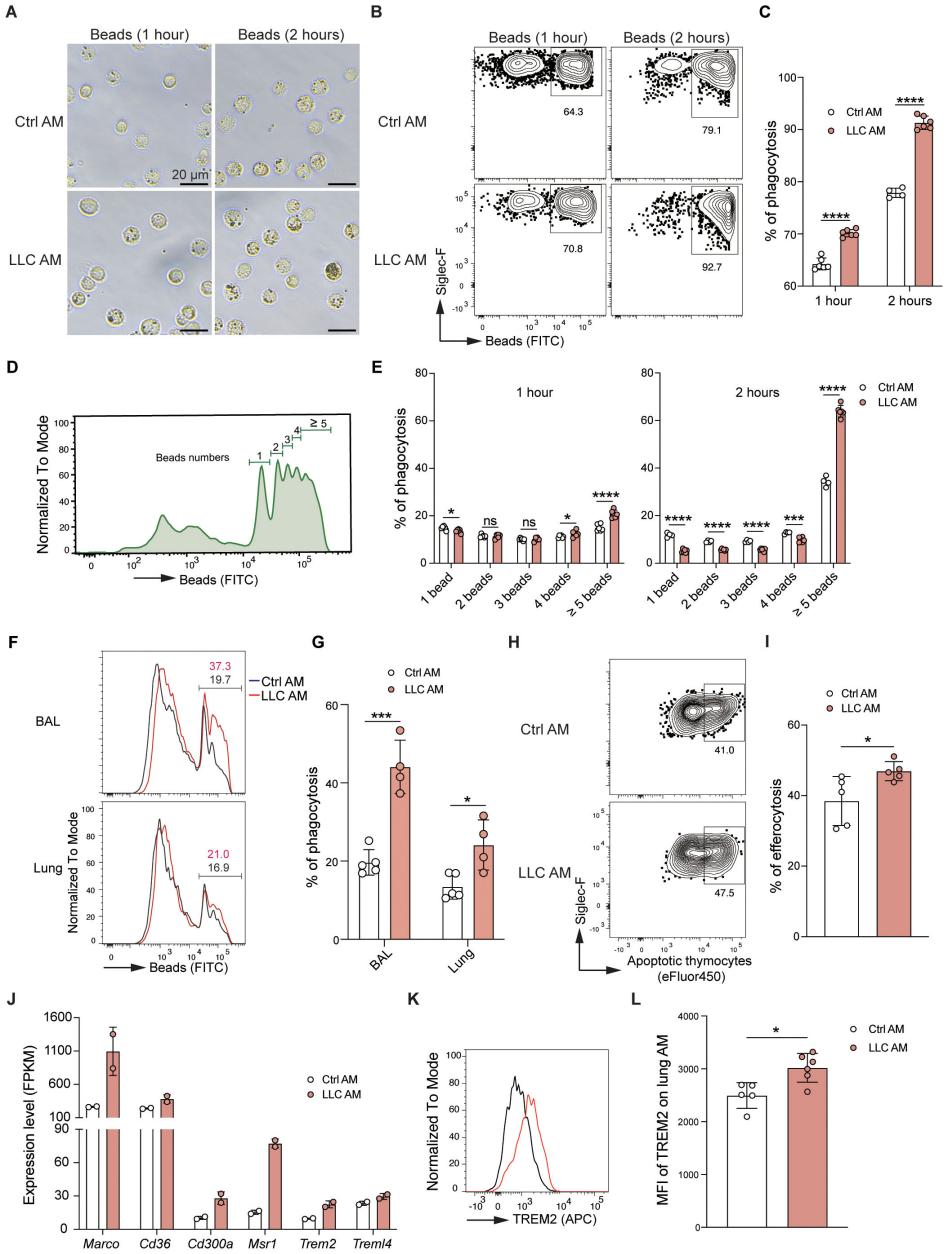
AMs exhibit enhanced phagocytic and efferocytotic capacity in the LLC-reprogrammed ETLME. **(A)** Representative microscopic images of Ctrl AM and LLC AM after incubation with fluorescein-labeled latex beads for 1 hour or 2 hours (Scale bar = 20 μm). **(B, C)** Flow cytometry analysis of the phagocytosis of Ctrl AM and LLC AM. Representative dot plots **(B)** and percentages **(C)** of AM phagocytosis. **(D)** Histogram showing the distribution of the number of beads phagocytosed by AM. **(E)** Quantification of the percentage of AM that engulf different numbers of beads (1 bead, 2 beads, 3 beads, 4 beads, and greater than or equal to 5 beads). **(F, G)** Fluorescein (FITC)-labeled latex beads were intratracheally (i.t.) delivered into the alveoli of control and tumor-bearing mice. Two hours later, flow cytometry analysis of bead uptake by AMs isolated from bronchoalveolar lavage (BAL) and lung tissues was performed. **(F)** The histograms represent the fluorescence intensity of the beads. **(G)** Percentages of beads positive AMs. **(H, I)** Flow cytometry analysis of Ctrl AM and LLC AM after incubation with fluorescein-labeled apoptotic thymocytes for 2 hours. Representative dot plots **(H)** and percentages **(I)** of AM efferocytosis. **(J)** Expression levels (FPKM) of phagocytosis- and efferocytosis-related genes (*Marco*, *Cd36*, *Cd300a*, *Msr1*, *Trem2*, and *Trem1l4*) in Ctrl AM and LLC AM. **(K)** Histogram showing the expression of TREM2 in Ctrl AM and LLC AM. **(L)** MFI of TREM2 on lung AM in Ctrl AM and LLC AM. The data are presented as mean ± SDs (n = 4–6 samples per group). Unpaired *t*-tests were used, “ns” indicates not significant, *P < 0.05, ***P < 0.001, and ****P < 0.0001.

Phagocytosis is a fundamental physiological function of AMs in the alveoli (10). AMs isolated from control and LLC-bearing mice were incubated *in vitro* with fluorescein-labeled latex beads, and phagocytosis events were assessed *via* microscopy (**Figure 6A**) and FACS (**Figures 6B-E**). Compared with control AMs, AMs from LLC-bearing mice presented higher percentages of fluorescein beads^+^ cells (**Figures 6A-C**), and AMs from LLC-bearing mice engulfed more beads at the individual cell level, as reflected by a greater percentage of AMs engulfing greater than or equal to 5 fluorescein beads (**Figures 6D, E**). To further evaluate phagocytic function *in situ*, fluorescein-labeled latex beads were intratracheally delivered into the alveoli. Consistent with the *in vitro* findings, AMs from LLC-bearing mice presented enhanced phagocytic capacity (**Figures 6F, G**). AMs are also important in the clearance of apoptotic cells through efferocytosis (31). Compared with control AMs, AMs incubated with fluorescent-labeled apoptotic thymocytes presented a greater percentage of efferocytosis in LLC-bearing mice (**Figures 6H, I**, **Supplementary Figure S5A**). Therefore, AMs exhibit enhanced phagocytic and efferocytotic functions in the LLC-created lung microenvironment, which is further supported by RNA sequencing data that revealed upregulated expression of genes associated with phagocytosis and efferocytosis, including *Marco*, *Cd36*, *Cd300a*, *Msr1*, *Trem2*, and *Treml4*, in AMs of LLC-bearing mice (**Figure 6J**) and increased protein expression of TREM2 (**Figures 6K, L**). Lipid metabolism is another key function of AMs (32). Oil Red O staining revealed no abnormal lipid accumulation in either control AMs or AMs of LLC-bearing mice (**Supplementary Figure S5B**), indicating that AMs maintain normal lipid metabolism function. Next, we tested the AM responsiveness to external stimuli. AMs were isolated and stimulated with LPS for 4 hours *in vitro*. Analysis of inflammatory cytokines in the supernatant revealed that control AMs and AMs from LLC-bearing mice secreted comparable levels of IL-1α and IL-6 after LPS stimulation, although AMs from LLC-bearing mice secreted slightly lower levels of TNF-α (**Supplementary Figure S6A**). PCA analysis of the transcriptome revealed that the difference between control AMs and AMs from LLC-bearing mice decreased after LPS stimulation (**Supplementary Figure S6B**). This finding was further supported by the GO enrichment analysis, as among the top 15 upregulated biological process gene clusters, there was an overlap of 10 gene clusters between them (**Supplementary Figure S6C**). Genes related to the toll-like receptor (**Supplementary Figure S6D**), TNF (**Supplementary Figure S6E**), and NF-kappa B (**Supplementary Figure S6F**) signaling pathways were similarly regulated in both control AMs and AMs from LLC-bearing mice upon LPS stimulation. These findings indicate that AMs in the LLC-reprogrammed ETLME do not lose the ability to respond to external stimuli. Altogether, these data demonstrate that AMs acquire enhanced phagocytic and efferocytotic functions while maintaining normal lipid metabolism and responsiveness to external stimuli in the LLC-reprogrammed ETLME.

### AMs gain enhanced anti-tumor activity in the LLC-reprogrammed ETLME

**Figure 7:**
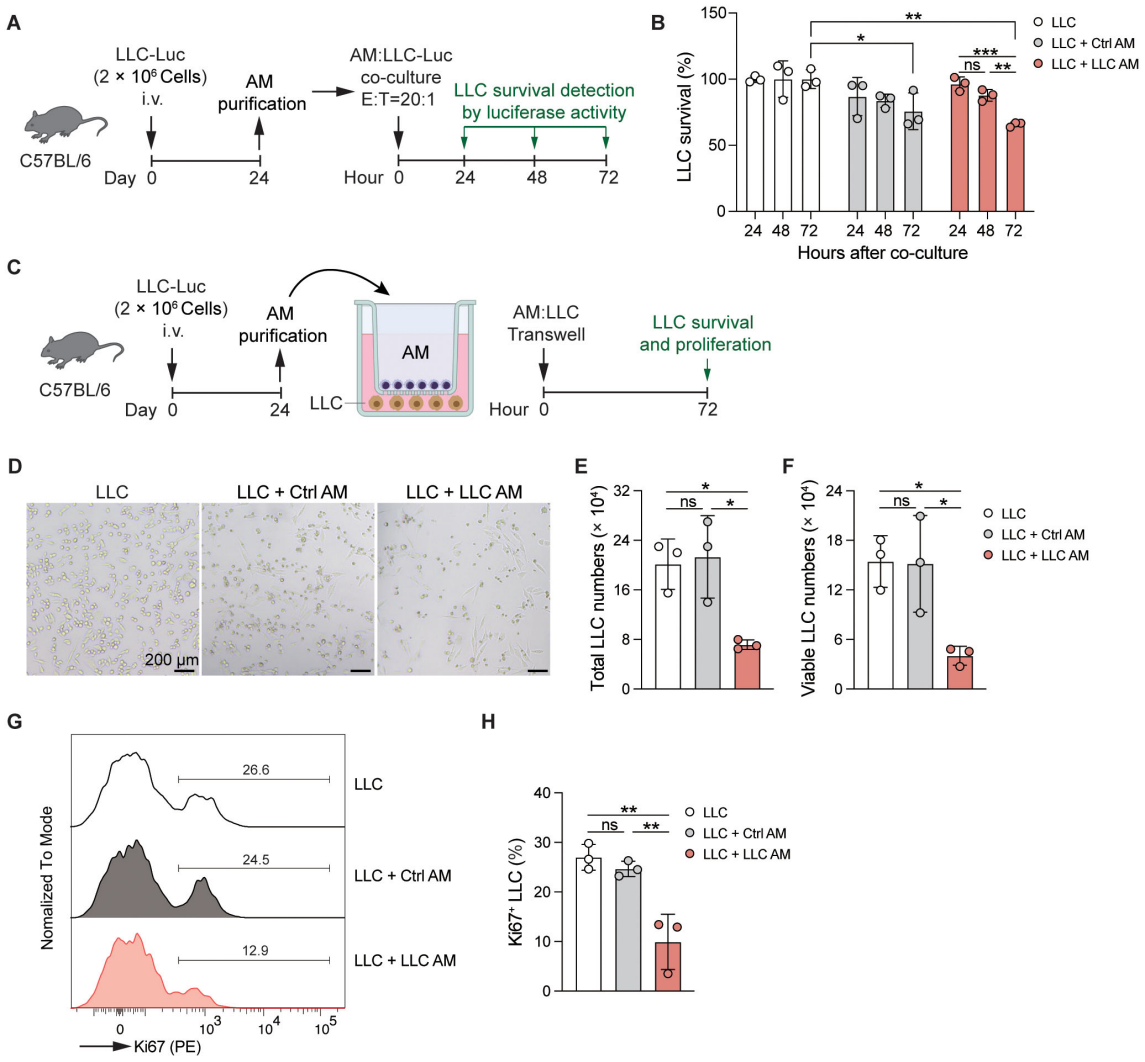
AMs gain enhanced anti-tumor activity in the LLC-reprogrammed ETLME. **(A)** Schematic of the experimental design. AMs were purified from BAL of LLC-Luc inoculated C57BL/6 mice at day 24 and co-cultured with LLC-Luc cells *in vitro* (E:T = 20:1). The survival of LLC was detected by luciferase activity of cell lysate at 24, 48, and 72 hours after co-culture. AMs isolated from untreated naïve mice were used as a control. **(B)** Survival of LLC co-cultured with or without Ctrl or LLC AM at indicated time points. **(C)** Schematic of the experimental design. AMs were purified from BAL of LLC-Luc inoculated C57BL/6 mice at day 24 and co-cultured with LLC-Luc cells in transwell *in vitro*. The survival and proliferation of LLC were detected at 72 hours after culture in D-H. AMs isolated from untreated naïve mice were used as a control. **(D-H)** Representative microscopic images **(D)**, total numbers **(E)**, viable numbers **(F)**, Representative Ki67 staining **(G)**, and percentage of Ki67^+^ cells of LLC in transwell culture for 72 h with or without Ctrl or LLC AM. The data are presented as mean ± SDs (n = 3 samples per group). Unpaired *t*-tests were used, “ns” indicates not significant, *P < 0.05, **P < 0.01, and ***P < 0.001.

Our experiments show that AMs are activated in the LLC-reprogrammed lung microenvironment and take on strengthened phagocytic and efferocytotic functions. To explore the anti-tumor activity of AMs in the lungs of LLC-bearing mice, AMs were isolated from the BAL of control or LLC-Luc inoculated C57BL/6 mice at day 24 and then co-cultured with LLC-Luc cells *in vitro* at an E:T ratio of 20:1 (**Figure 7A**). After 72 hours, the survival of LLC cells was found to be decreased in both the control AM and LLC AM co-cultured groups. Notably, in the LLC AM co-cultured group, the survival of LLC cells declined progressively over time (**Figure 7B**). These findings indicate that AMs possess anti-tumor activity *in vitro* when in direct contact with tumor cells. However, as AMs are not typically recruited to the tumor lesions, direct contact between AMs and tumor cells is not a common occurrence *in vivo*. To determine whether soluble factors might mediate this anti-tumor effect, AMs were again purified from the BAL of control or LLC-Luc inoculated C57BL/6 mice at day 24 and co-cultured with LLC-Luc cells in a transwell setup *in vitro* at an E:T ratio of 1:1 (**Figure 7C**). After 72 hours of culture, both the total numbers and viable counts of LLC cells were significantly reduced in the LLC AM co-cultured group compared to the LLC only group and the control AM co-cultured group (**Figures 7D-F**). Moreover, the percentage of Ki67^+^ LLC cells was also decreased in the LLC AM co-cultured group (**Figures 7G, H**), indicating the limited proliferation of LLC cells. These results suggest that LLC-experienced AMs acquire the ability to restrict tumor growth in a non-contact manner.

## Discussions

This study examined how the lung tumor-created microenvironment affects AMs by investigating their distribution, phenotype, and functional adaptations. Our findings showed that, while LLC tumors induce notable changes in the lung immune landscape, AMs preserve their tissue localization and essential physiological functions and gain enhanced anti-tumor activity.

Analysis of immune cells in tumor-bearing lungs revealed that LLC promotes lung inflammation, remodels the lung immune cell composition, and induces an exhaustion phenotype of T cells. Further examination of large tumor nodules versus remaining lung tissues revealed that significant immune cell compositional shifts occurred predominantly in tumor lesions, with minimal changes in unaffected regions. These findings demonstrate that the TME and the ETLME are functionally and spatially distinct. Consequently, the spatial positioning of AMs is critical in determining their phenotypic and functional states.

One of the most interesting observations of our study was that AMs remained resident in the lung tissues and were rarely found within tumor lesions in the LLC mouse model, indicating that AMs are spatially segregated from the TME. The possible reason would be that AMs locate the alveolar spaces in homeostasis, which are spatially separated from the interstitium and blood vessels by the alveolar-capillary barrier. Therefore, when tumor cells are introduced into the lung *via* intravenous injection, AMs do not directly interact with tumor cells. However, these results contrast with those of previous study, which demonstrated that influenza-trained AMs could infiltrate lung tumor lesions and exert sustained anti-tumor effects in an intravenously injected B16F10 lung tumor (33). The differential AM localization patterns may reflect distinctions between tumor models. As B16F10 tumor cells are known to be more invasive and capable of forming spontaneous metastases in mouse models, the increased infiltration of AMs into B16F10 lung lesions may result from compromised alveolar structures. Some lung tumors originate from alveolar epithelial cells, arise directly within the alveolar spaces. In such cases, AMs would naturally coexist in the same anatomical compartment as the tumor cells, leading to a different pattern of interaction. The distinct AM localization patterns in different tumor models are worth exploring further in the future.

Our data demonstrated that AMs do not serve as precursors for TAMs in LLC tumors, which play crucial roles in establishing immunosuppressive TME. In large LLC tumor nodules, the predominant TAM population was CD11b^+^ macrophages, whose proportions and absolute numbers were significantly greater in tumors than in adjacent lung tissues. The special distribution of AMs allows their functional ability maintenance, but they were not reprogrammed by the TME.

Unlike the TME, which has an immunosuppressive phenotype, the ETLME generally shows immune activation. Consistently, AMs in the ETLME displayed an enhanced dynamic equilibrium between cell death and proliferation. This enhanced maintenance capacity of AMs in LLC-bearing lungs mirrors observations in other models of lung perturbation, including adenoviral infection (34), influenza infection (35), and low-dose irradiation (36), suggesting a conserved mechanism for AM pool maintenance under various stress conditions.

In addition to their capacity for proliferation, AMs also undergo significant modifications, including altered gene expression profiles, enhanced phagocytic capacity and anti-tumor activity, suggesting tumor-reprogrammed lung microenvironment mediates this modulation of their functional capabilities. However, which environmental and intrinsic factors that are critical to lead to this significant modulation for AMs are not well understood. To find out the key cytokines and metabolites in the lungs and key genes in AMs induced by lung tumor growth would be important to understand this process.

While our study provides valuable insights into the behavior of AMs in the LLC-created lung microenvironment, several important questions warrant further investigation. First, the potential involvement of AMs in modulating immunotherapy responses remains unexplored and represents a critical area for future research. Second, it is essential to determine whether the functional adaptations we observed in AMs are specific to the LLC model or represent a conserved response across different lung cancer subtypes. Comparative studies using alternative tumor models could help establish the broader relevance of these findings.

AMs have been reported to profoundly influence anti-tumor immune responses in the lungs. After pre-training for respiratory influenza infection, AMs infiltrate into the lung tumor lesions and exhibit long-lasting anti-tumor effects through enhanced phagocytic and tumor cell cytotoxic capacity (33). AMs support the progression of lung cell carcinoma by producing activin A. Using an antagonist of activin A can inhibit the proliferation of lung cancer cells, and depletion of activin A during the postnatal period can also limit tumor growth in the lungs (37). Thus, understanding which factors in tumor-reprogrammed microenvironments are important for anti-tumor activity of AMs will facilitate the development of therapies for lung cancer based on AMs (38). Furthermore, increasing proliferation and enhancing functions by targeting *in situ* AMs or the adoptive transfer of function-enhanced AMs would be potential strategies for lung cancer in the future.

In summary, the growth of LLC disrupts the homeostatic immune microenvironment, generating two functionally and spatially different immune microenvironments in the lungs, each with a unique immune cell composition. Within TME, LLC establishes an immunosuppressive microenvironment characterized by the accumulation of CD11b^+^ tumor-associated macrophages and neutrophils, alongside a predominance of T cells with exhaustion phenotype. In contrast, the ETLME exhibits an immune-activated/inflammatory microenvironment, featuring a small amount of lymphocyte and CD11b^+^ macrophage infiltration. Here, AMs increase their proliferation capacity to counterbalance inflammation-induced cell death. Notably, AMs maintain their tissue localization without migrating into tumor lesions. In tumor-bearing lungs, AMs adopt an activated phenotype, marked by upregulation of CD11b, downregulation of Siglec-F expression, elevated expression of inflammatory genes, and enhanced phagocytic, efferocytotic, and anti-tumor activity (**Supplementary Figure S7**).

## Materials and methods

### Mice

WT female C57BL/6 mice were purchased from GemPharmatech. Mice were housed in a specific pathogen-free facility and were maintained on a 12-hour light/dark cycle with 50-60% humidity at 20-25°C. The animals were assigned to experimental groups at random. Female mice aged 6–8 weeks were used for all the experiments, and the numbers of mice per experimental group are indicated in the figure legends. All experiments were performed following the guidelines of the Experimental Animal Welfare and Ethics Committee of the Institute of Health and Medicine, Hefei Comprehensive National Science Center. The project is approved for implementation with the approval number IHM-AP-2024-059.

### LLC-mouse models

The mice were intravenously injected with 2×10^6^ luciferase-expressing syngeneic murine Lewis Lung Cancer cells (LLC-Luc) in 200 µL of PBS. The tumor cell nodules formed in the lung were measured via *ex vivo* luciferase-based non-invasive bioluminescence imaging using IVIS Lumina II (PerkinElmer) or H&E staining of lung paraffin sections.

### Mouse lung tissue processing and BAL cell collection

The mice were euthanized with 400 mg/kg pentobarbital sodium by intraperitoneal injection. BAL fluid was obtained by flushing 3 times with 400 µL of ice-cold PBS (containing 2 mM EDTA) through an intratracheal cannula. The cells in the BAL were collected *via* centrifugation for flow cytometry analysis and staining. The BAL supernatants were collected for the detection of cytokines and chemokines, protein concentration, and turbidity. After BAL collection, the right ventricle of the heart was gently perfused with 10 mL of ice-cold 1×PBS to remove blood cells. The lung tissues were collected and cut into small pieces and digested with IMDM (Thermo Fisher Scientific, C12440500BT) containing 0.5 mg/mL type IV collagenase (Sigma Aldrich C5138), 0.05 mg/mL DNase I (Roche 10104159001), and 3% FCS at 37 °C and 130rpm in a shaker for 45 min. A single-cell suspension was obtained after red blood cell lysis with ammonium-chloride-potassium (ACK) lysis buffer, passed through a 70-µm cell strainer, and washed with fluorescence-activated cell sorting (FACS) buffer (PBS containing 2% FCS and 2 mM EDTA). A single-cell suspension was centrifuged and resuspended in the appropriate buffer for flow cytometry analysis.

### Luciferin-based *in vivo* tumor imaging

The mice were anesthetized with isoflurane and injected intraperitoneally (i.p.) with 150 mg/kg d-luciferin (Absin, 42075819). After injection, the mice were placed on the imaging platform of an IVIS Spectrum imaging station (PerkinElmer) with continuous isoflurane inhalation. White light and luciferase activity were recorded for 40 s starting at 8 min after the d-luciferin injection. Living Image software (PerkinElmer) was used for luciferase activity quantification.

### Lung histopathology

After cardiac perfusion, the lungs were removed and fixed in 4% neutral formaldehyde. The fixed lung tissues were hydrated in running water and dehydrated in ethanol with gradually increasing concentrations (50% 15 min,70% 15 min, 70% 15 min, 80% 30 min, 95% 30 min, 100% 15 min, 100% 15 min). The tissue was cleared in xylene and then soaked in wax. The tissue was embedded in wax blocks and cut into 5 μm sections, which were deparaffinized and hydrated. After hydration, the sections were stained with hematoxylin for 5 min, washed away with running water to remove the floating color, stained with eosin for 5 min, washed with running water, dehydrated and made transparent. The sections were then sealed with neutral gum, air-dried, observed under a microscope, and photographed. Follow-up scanning with TissueFAXS Plus S.

### Lung immunofluorescence staining and imaging

Frozen lung tissue sections were permeabilized at room temperature using 0.3% Triton X-100 in PBS for 20 minutes. The sections were then blocked with a solution containing 2% BSA, 0.3% Triton X-100, and 10% normal goat serum in PBS for 1 hour. After blocking, the sections were incubated with primary antibodies, anti-mouse Siglec-F PE/Dazzle 594 (clone S17007L, Biolegend) and anti-mouse CD11c Alexa Fluor^®^ 647 (clone N418, Biolegend) (antibodies are listed in **Table 1**), overnight at 4°C. The following day, the sections were stained with DAPI (Sigma, D9542) at room temperature for 10 minutes. Microscopy was performed using a confocal microscope (Zeiss LSM980), and ImageJ software was used for quantification.

**Table 1:** Information for fluorescent dyes and antibodies.

Name	Clone	Supplier	Cat. No.	Concentration	RRID	Dilution
PE/Dazzle 594 anti-mouse CD24	M1/69	Biolegend	101838	0.2 mg/ml	AB_2566732	1:3000
PE/Cy7 anti-mouse CD19	1D3	BD Pharmingen	552854	0.2 mg/ml	AB_394495	1:1000
PE/Cy7 anti-mouse Tim-3	RMT3-23	Biolegend	119715	0.2 mg/ml	AB_2571932	1:200
PE anti-mouse TCR γ/δ	UC7-13D5	Biolegend	107507	0.2 mg/ml	AB_345265	1:400
PE anti-mouse Ki-67	16A8	Biolegend	652404	0.2 mg/ml	AB_2561525	1:500
FITC anti-mouse CD335 (Nkp46)	29A1.4	Biolegend	137606	0.5 mg/ml	AB_2298210	1:400
FITC anti-mouse CD44	IM7	Biolegend	103006	0.5 mg/ml	AB_312957	1:400
FITC anti-mouse MHC-II	M5/114.15.2	Biolegend	107606	0.5 mg/ml	AB_313321	1:1000
BV785 anti-mouse CD45	30-F11	Biolegend	103149	0.2 mg/ml	AB_2564590	1:1000
BV785 anti-mouse CD62L	MEL-14	Biolegend	104440	0.2 mg/ml	AB_2629685	1:4000
BV711 anti-mouse CD11c	N418	Biolegend	117349	0.5 mg/ml	AB_2563905	1:3000
BV605 anti-mouse CD11b	M1/70	Biolegend	101257	0.2 mg/ml	AB_2565431	1:800
BV421 anti-mouse TCRβ	H57-597	Biolegend	109230	0.2 mg/ml	AB_2562562	1:800
BV421 anti-mouse PD-1	RMP1-30	Biolegend	109121	0.2 mg/ml	AB_2687080	1:200
BV421 anti-mouse CD170 (Siglec-F)	S17007L	Biolegend	155509	0.2 mg/ml	AB_2810421	1:400
APC-Cy7 anti-mouse Ly6C	HK1.4	Biolegend	128026	0.2 mg/ml	AB_10640120	1:3000
APC/Cy7 anti-mouse CD4	GK1.5	BD Pharmingen	552051	0.2 mg/ml	AB_394331	1:1000
APC anti-mouse CD3	17A2	Biolegend	100236	0.2 mg/ml	AB_2561456	1:1000
APC anti-mouse TREM-2	237920	RD-Systems	FAB17291A	0.5 mg/mL	/	1:100
APC anti-mouse CD64	X54-5/7.1	Biolegend	139306	0.2 mg/ml	AB_11219391	1:800
Alexa Fluor^®^ 700 anti-mouse CD8a	53-6.7	Biolegend	100730	0.5 mg/ml	AB_493703	1:1000
Alexa Fluor^®^ 700 anti-mouse Ly6G	1A8	Biolegend	127622	0.5 mg/ml	AB_10643269	1:3000
BV650 anti-mouse F4/80	BM8	Biolegend	123149	0.2 mg/ml	AB_2564589	1:200
PE/Dazzle 594 anti-mouse CD170 (Siglec-F)	S17007L	Biolegend	155530	0.2 mg/mL	AB_2890716	1:200
Alexa Fluor^®^ 647 anti-mouse CD11c	N418	Biolegend	117312	0.5 mg/mL	AB_389328	1:200
PE Annexin V	/	Biolegend	640908	12ug/ml	AB_2561298	1:400
Zombie Aqua™ Fixable Viability Kit	/	Biolegend	423102	0.5 mg/ml	/	1:1000
Fixable Viability Stain 780 (FVS780)	/	BD Pharmingen	565388	1.11mg/ml	AB_2869673	1:2000
Cell Proliferation Dye eFluor™ 450	/	Thermo Fisher Scientific	65-0842-85	/	/	/

### Cytokine and chemokine analysis

The upper right lobe of the lungs was removed and placed into Eppendorf tubes with 100 μL sterile PBS and two 4-mm steel balls and then ground at 60 HZ for 70 s (JXFSTPRP-CLN-24L). The supernatant of the lung tissue homogenate was collected and stored in a -80°C freezer. Cytokine and chemokine levels in BAL fluid and lung tissue homogenate were determined *via* the LEGENDplex™ mouse inflammation panel with a V-bottom Plate (Biolegend Cat. No.740446).

### Flow cytometry

FACS analysis was performed using LSRFortessa (BD Biosciences) and CytoFLEX LX (Beckman), and cell sorting was performed using Beckman CytoFLEX SRT (Beckman). The data were analyzed with FlowJo software (TreeStar). Single-cell suspensions from BAL and lungs were plated in V-bottom 96-well plates in PBS. The cells were stained with a Zombie Aqua fixable viability kit for 15 min and then washed and incubated in FACS buffer containing 0.5% rat serum for 15 min on ice for Fc blocking. The cells were then washed and stained with fluorochrome-labeled antibodies (listed in **Table 1**) for 30 min in FACS buffer. The cells were subsequently washed twice with FACS buffer before detection.

For cell death detection, the cells were stained with Fixable Viability Stain 780 (FVS780, BD Pharmingen, 565388) for 15 min and then stained with PE-Annexin V (Biolegend, 640908) in 50 uL of 1× Annexin V Binding Buffer (BD Pharmingen, 556454) for 10 min, then 150 uL 1× Annexin V Binding Buffer was added, and the cells were directly detected without washing.

For the intracellular staining of PE-conjugated anti-mouse Ki-67, after the cell surface marker was stained as described above, the cells were fixed and permeabilized with Foxp3/Transcription Factor Staining Buffer Set (Thermo Fisher, 00-5523-00). In brief, the cells were resuspended in Fixation/permeabilizing buffer for 45 min and then washed twice with permeabilizing buffer, followed by intracellular staining with anti-Ki67 diluted in permeabilization buffer for 30 min at room temperature in the dark. The cells were washed twice with permeabilization buffer and twice with FACS buffer before detection.

### BCA protein and turbidity detection

The BCA protein assay kit was obtained from Thermo Scientific (Cat. No. 23225). A total of 25 µL of each BAL sample was used. VERSAmax microplate reader (Molecular Devices) was used for colorimetric quantification and analysis at a wavelength of 562 nm. For the turbidity detection, 250 µL of BAL fluid was transferred into a flat-bottom 96-well plate, and the optical density (OD) was measured at 600 nm using a Microplate reader (PerkinElmer, EnSight).

### PKH26 labeling of AMs

The PKH26 phagocytic cell labeling kit (Sigma, PKH26PCL) was employed following the manufacturer’s instructions. A 5 μM PKH26 dye solution was prepared in Diluent B for *in vivo* experiments. Mice were intratracheally administered with 50 μL of the PKH26 dye solution one day before LLC tumor inoculation. On day 24 post-tumor inoculation, large tumor nodules and lung tissues were separately harvested. Immune cells were then isolated, and PKH26-labeled AMs were detected by flow cytometry.

### Phagocytosis assay for AMs

Tumor-bearing and control mice were sacrificed by intraperitoneal injection of an overdose of 1% pentobarbital sodium (400 mg/kg). For *in vitro* phagocytosis detection, BAL samples were collected, and AMs were counted by microscopy (large cells). The cells were resuspended in DMEM medium, and the cells containing 1×10^5^ AMs were plated in 24-well plates at a density of 1mL/well. After incubating at 37 °C for 30 minutes, the floating cells were removed. 1mL of DMEM medium containing 10% FBS with 0.5 uL (1:2000 dilution) of fluorescent latex beads (Sigma-Aldrich, Cat. No. L4655, 1.0 μm) were added to AMs. After 1 or 2 hours, free beads were removed from the wells, and AMs were washed with PBS for subsequent microscopy. Then AMs were collected from wells with PBS containing 4 mM EDTA and used for the next step of flow cytometry detection. For *in vivo* phagocytosis detection, the mice were anesthetized by intraperitoneal injection of 1% pentobarbital sodium (80–100 mg/kg), after which 100 μL of 1:1000 diluted fluorescent latex beads (Sigma-Aldrich, Cat. No. L4655, 1.0 μm) in PBS were intratracheally (i.t.) delivered to the mice. After 2 hours, mice were sacrificed and the phagocytosis of AMs from the BAL and lungs was detected *via* flow cytometry.

### Preparation of apoptotic thymocytes and efferocytosis assay for AMs

Thymuses were harvested from the mice and mechanically dissociated into single-cell suspensions. The suspensions were centrifuged at 500 × g for 5 min, and the supernatant was discarded. The cells were resuspended in 3 mL of DMEM medium containing 30 μL of dexamethasone (ZoFtic, ZF341, 10 mM) and incubated for 24 h in a humidified chamber (37°C, 5% CO_2_). After incubation, the cells were collected, centrifuged (500 × g, 5 min), and washed to remove the supernatant. The cells were subsequently stained with 3 μg/mL Cell Proliferation Dye eFluor™ 450 (Thermo Fisher Scientific, 65-0842-85) for 15 min at room temperature (protected from light). Following staining, the cells were washed twice and resuspended in DMEM medium at a density of 5 × 10^5^ cells/mL, and then kept on ice until use. For *in vitro* efferocytosis detection, AMs were resuspended in DMEM at 1 × 10^5^ cells/mL and seeded into a 24-well plate (1 mL/well). After 30 min of incubation, floating cells were removed. Stained apoptotic thymocytes (1 mL/well) were added to a 24-well plate and co-cultured with AMs for 2 hours. After incubation, the supernatant was discarded, and the cells were gently washed once with PBS. Then, 500 μL of PBS was added to each well for imaging. For further analysis, 0.25% trypsin-EDTA was added, the cells were incubated at 37°C for 10 min, and then the cells were collected for flow cytometry.

### LPS stimulation of AMs

BAL fluid was collected from the tumor-bearing and control mice. AMs were resuspended at a density of 1×10^5^ cells/mL in complete DMEM (supplemented with 10% FCS), seeded into a 24-well plate (1 mL/well). Non-adherent cells were then removed after incubating for 30 min. AMs were stimulated for 4 hours with DMEM medium containing 400 ng/mL lipopolysaccharide (LPS) from *E. coli* 055:B5 (InvivoGen, tlrl-b5lps) or in LPS-free medium (as control) for 4 hours. After stimulation, the supernatant was collected for cytokine/chemokine analysis. The cells were lysed in 1 mL of TRIzol for subsequent RNA extraction and sequencing.

### RNA sequencing

The indicated AMs (1×10^5^ cells) were used for bulk RNA sequencing. Total RNA was extracted with a miRNeasy Micro Kit (Qiagen). Total RNA (more than 100 ng) was used for the subsequent library preparation using the VAHTS Universal V8 RNA-seq Library Prep Kit (Illumina). Poly(A) mRNA isolation was performed using Oligo(dT) beads. The mRNA fragmentation was performed using divalent cations and high temperature. Priming was performed using random primers. First-strand cDNA and the second-strand cDNA were synthesized. The purified double-stranded cDNA was then treated to repair both ends and dA-tailing was added in one reaction, followed by a T-A ligation to add adaptors to both ends. Size selection of adaptor-ligated DNA was then performed using DNA clean beads. Each sample was then amplified by PCR using P5 and P7 primers, and the PCR products were validated. Libraries with different indexes were then multiplexed and loaded on an Illumina NovaSeq instrument for sequencing using a 2×150 paired-end configuration according to the manufacturer’s instructions. The fragments were mapped to the ensemble mouse reference genome GRCm39 (version 2020) using the STAR aligner. Differential expression analysis (DESeq2) identified significant genes (fold change > 2 or < -2, p < 0.05).

### Oil red O staining of AMs

AMs (3×10^4^ cells) were resuspended in 200 μL of PBS containing 2 mM EDTA and were then cytocentrifuged onto glass slides (2000 rpm, 5 min). After air-drying, the slides were fixed with 4% paraformaldehyde. The slides were subsequently rinsed with 70% ethanol to remove residual fixative. The samples were then immersed in Oil Red O staining solution (Servicebio, G1015) for 5–15 min with gentle agitation to ensure uniform dye distribution. After staining, the excess dye was washed off with 60% isopropanol, followed by a 30-second rinse with running water. The slides were counterstained with hematoxylin for 2 min (to avoid prolonged exposure to prevent overstaining of the nuclei) and thoroughly rinsed under running water to remove residual hematoxylin. Finally, the slides were mounted with glycerol gelatin, minimizing air exposure to prevent fading, and care was taken to ensure flat positioning without air bubbles. Microscopic examination revealed lipid droplets as deep orange-red or bright red, whereas nuclei appeared blue-purple. The entire stained sections were scanned using a Tissue FAXS Plus S microscope for comprehensive analysis.

### Antitumor activity assay for AMs

For direct co-culture experiments of AMs and tumor cells, AMs from LLC-bearing or naive mice were isolated and seeded in a 96-well plate at a density of 1 × 10^5^ cells per well. The cells were incubated in a cell culture incubator (37°C and 5% CO_2_) for 2 hours. Subsequently, LLC-Luc tumor cells were seeded in AM culture wells at 5 × 10^3^ cells per well. At 24, 48, and 72 hours after co-culture, the cells were lysed, and luciferase activity per well was measured using a luciferase assay system (Yeason Biotechnology) in accordance with the manufacturer’s instructions. Luminescence measurements were conducted on an EnSight Multimode Plate Reader (Perkin Elmer). Tumor cell survival was calculated and presented as a percentage of normalized luminescence relative to LLC-only culture wells. In transwell co-culture experiments, LLC-Luc tumor cells were cultured in 24-well tissue culture plates at a density of 1 × 10^5^ cells per well in 500 uL of medium, AMs at a density of 1 × 10^5^ cells per well in 100 uL, cultured with 30 ng/mL of GM-CSF, were separated from LLC-Luc tumor cells using transwell inserts with a pore size of 0.4 μm. At 72 hours after co-culture, images of LLC-Luc cells were captured *via* bright field imaging. Subsequently, the cells were collected, and cell numbers were counted. Cell proliferation was analyzed through Ki67 staining.

### Statistical analysis

Data are presented as the mean ± SDs and were analyzed with GraphPad Prism software. Unpaired t-tests (two-group comparisons) and one-way ANOVA (multiple group comparisons) were used for statistical analysis. “ns” = not significant; *P < 0.05, **P < 0.01, ***P < 0.001, and ****P < 0.0001.

## Data Availability

The RNA-seq data are available at the Gene Expression Omnibus (GEO) repository under the accession number GSE301919.
