# Supplementary material for: Alveolar macrophages maintain tissue localization and gain enhanced anti-tumor activity in Lewis lung carcinoma-reprogrammed lung microenvironment
Source: Front Immunol. 2025 Jul 24;16:1616514. doi: 10.3389/fimmu.2025.1616514 (PMC12328318; doi:10.3389/fimmu.2025.1616514)
Supplement: Supplementary file 1 [file DataSheet1.docx]

**Alveolar Macrophages Maintain Tissue Localization and Gain Enhanced Anti-Tumor Activity in Lewis Lung Carcinoma-Reprogrammed Lung Microenvironment**

Mengfei Ren^1,2#^, Jiaxiang Dou^2#^, Qian Yue^1,2^, Liqin Ma^1,2^, Hang Yu^2,3^, Shengwen Shang^2,4^, Shijie Wang^5,6^, Jian Wang^2,5,6,7*^, Tingting Li^8*^, Fengqi Li^1,2,3,4,6*^

^1^School of Basic Medical Sciences, Center for Big Data and Population Health of IHM, Anhui Medical University, Hefei, 230032, China

^2^Institute of Health and Medicine, Hefei Comprehensive National Science Center, Hefei, China

^3^Center for Xin’an Medicine and Modernization of Traditional Chinese of IHM, Anhui University of Chinese Medicine Hefei, 230012, China

^4^School of Biomedical Sciences and Engineering, South China University of Technology, Guangzhou International Campus, Guangzhou, 511442, China

^5^Department of Neurology, The First Affiliated Hospital of USTC, Division of Life Sciences and Medicine, University of Science and Technology of China, Hefei, China

^6^State Key Laboratory of Immune Response and Immunotherapy, Division of Life Sciences and Medicine, University of Science and Technology of China, Hefei, China

^7^Shanxi Province Cancer Hospital, Shanxi Hospital Affiliated to Cancer Hospital, Chinese Academy of Medical Sciences, Cancer Hospital Affiliated to Shanxi Medical University, Taiyuan, China

^8^Department of Clinical Laboratory, First Affiliated Hospital of Anhui Medical University, Hefei, Anhui, China

^#^These authors contributed equally to this work.

^*^Correspondence:

Jian Wang, ustcwj@ustc.edu.cn

Tingting Li, litingting_0725@126.com

Fengqi Li, [fengqi.li@ihm.ac.cn](mailto:fengqi.li@ihm.ac.cn)

**Supplementary figures and figure legends**


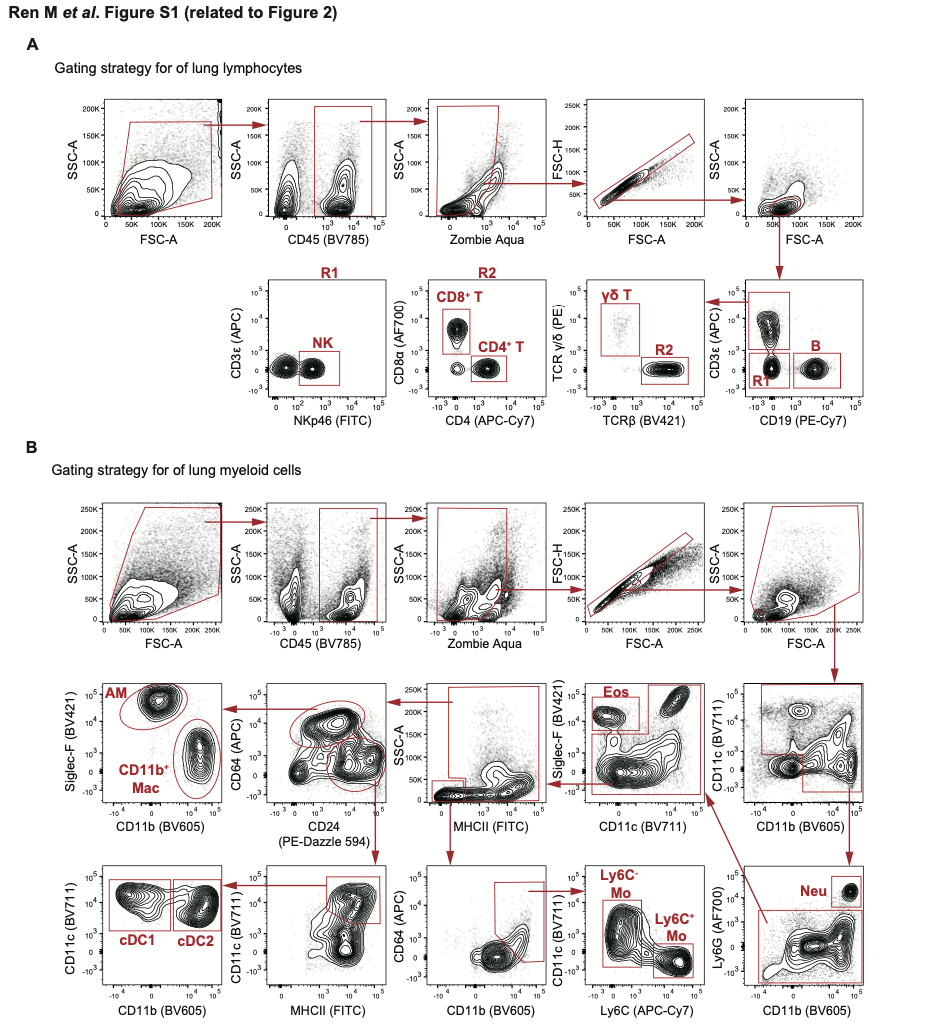


**Figure S1. Gating strategy for lung lymphocytes and myeloid cells (related to Figure 2).**

(A) Gating strategy for lung lymphocytes. Sequential plots illustrating the identification and separation of different lymphocyte subsets. First, the cells are selected based on the forward-scatter area (FSC-A) and side-scatter area (SSC-A). Then, CD45^+^ immune cells are gated, dead cells are excluded *via* Zombie Aqua staining, and doublets are excluded using FSC-A and FSC-H. Further gating was performed to distinguish natural killer (NK) cells (CD19⁻CD3ε⁻NKp46⁺), CD8⁺ T cells (CD19⁻CD3ε⁺ TCRβ^+^CD8α⁺CD4⁻), CD4⁺ T cells (CD19⁻CD3ε⁺TCRβ^+^CD8α⁻CD4⁺), γδ T cells (CD19⁻CD3ε^+^TCRβ⁻TCRγδ⁺), and B cells (CD19⁺CD3ε⁻). (B) Gating strategy for lung myeloid cells. The sequential plots depict the identification and separation of different myeloid cell subsets. First, CD45^+^ alive singlets are gated. Neutrophils (Neu) are identified as CD11b^+^Ly6G^+^ cells. Eosinophils (Eos) are identified as Ly6G^-^Siglec-F^+^CD11c^-^ cells. Alveolar macrophages (AMs) were identified as Ly6G^-^CD64^+^Siglec-F^hi^CD11b^-^ cells. CD11b^+^ macrophages were identified as Ly6G^-^CD64^+^Siglec-F^-^CD11b^+^ cells. Dendritic cells (DCs) are identified as Ly6G^-^CD24^+^CD64^-^MHCII^hi^CD11c^hi^ cells, and cDC1 and cDC2 are distinguished by CD11b. Monocytes are identified as Ly6G^-^MHCII^hi^CD64^lo^CD11b^+^ cells, and Ly6C^+^ Mo and Ly6C^-^ Mo are distinguished by CD11c and Ly6C.

**
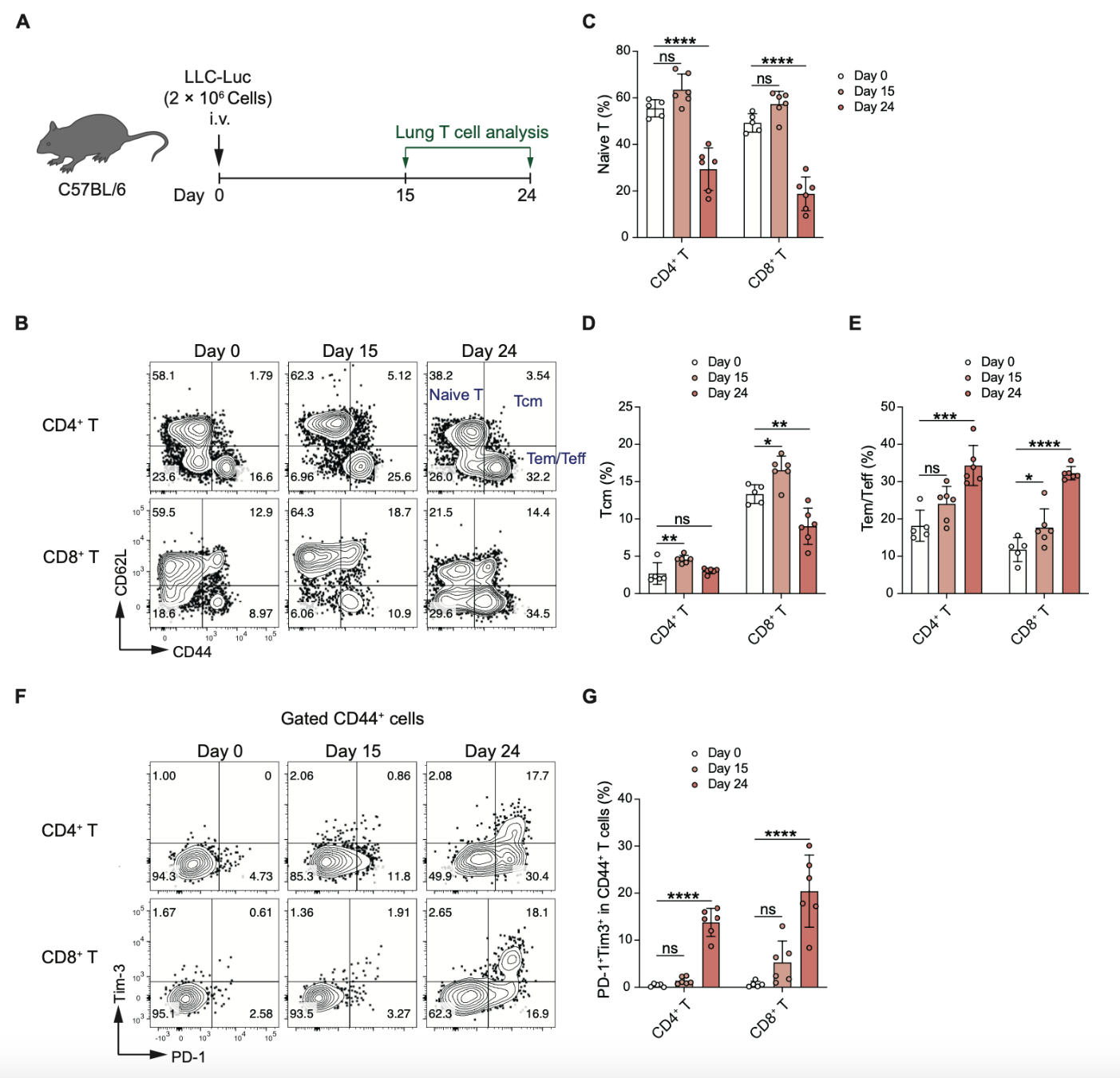
**

**Figure S2. LLC tumor growth promotes lung T-cell exhaustion (related to Figure 2).**

(A) Schematic of the experimental design. LLC-Luc were intravenously (i.v.) inoculated into C57BL/6 mice, then lung T cells were analyzed at days 0, 15, and 24. (B-G) Flow cytometric analysis of CD4⁺ and CD8⁺ T cells. (B) Representative dot plots showing the distributions of naive T cells, central memory T cells (Tcm), and effector memory T cells/effector T (Tem/Teff) cells based on CD62L and CD44 expression. (C-E) Percentages of naive T cells (C), Tcm (D), Tem/Teff (E) in CD4⁺ and CD8⁺ T cells. (F) Representative dot plots showing the expression of the inhibitory receptors PD-1 and Tim-3 on CD44⁺CD4⁺ and CD44⁺CD8⁺ T cells. (G) Percentages of PD-1^+^ Tim-3^+^ cells among CD44⁺CD4⁺ and CD44⁺CD8⁺ T cells. The data are presented as means ± SDs (n = 5-6 mice per group). One-way ANOVA was used, "ns" indicates not significant, **P* < 0.05, ***P* < 0.01, ****P* < 0.001, and *****P* < 0.0001.


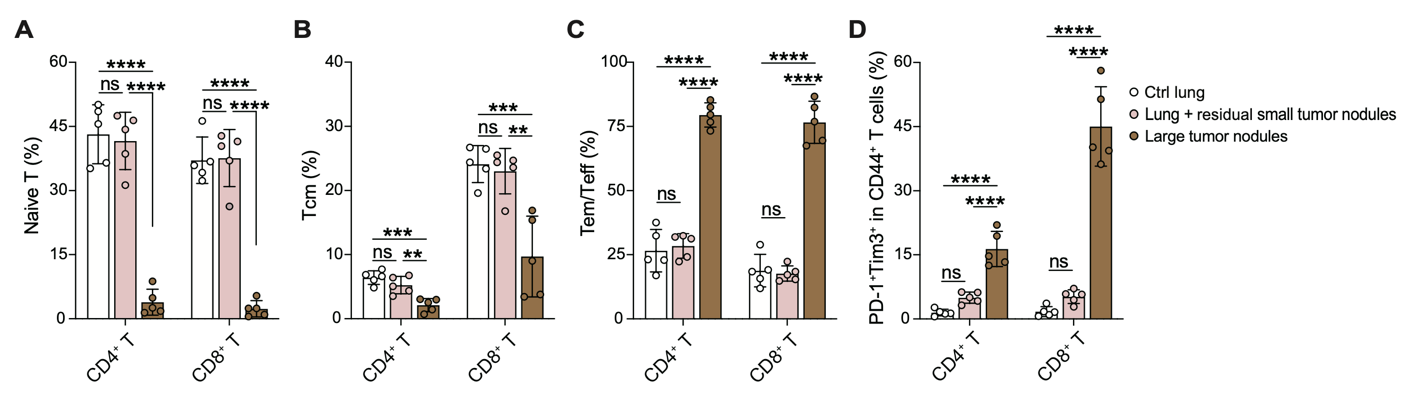


**Figure S3. T cells with an exhaustion phenotype appeared mainly in LLC tumor nodules (related to Figure 3).**

The experiments were performed as described in Figure 3. (A-C) Percentages of naive T cells (A), Tcm (B), and Tem/Teff (C) in CD4⁺ and CD8⁺ T cells. (D) Percentages of PD-1^+^Tim-3^+^ cells in CD44⁺CD4⁺ and CD44⁺CD8⁺ T cells. The data are presented as means ± SDs (n = 5 mice per group). One-way ANOVA was used, "ns" indicates not significant, ***P* < 0.01, ****P* < 0.001, and *****P* < 0.0001.


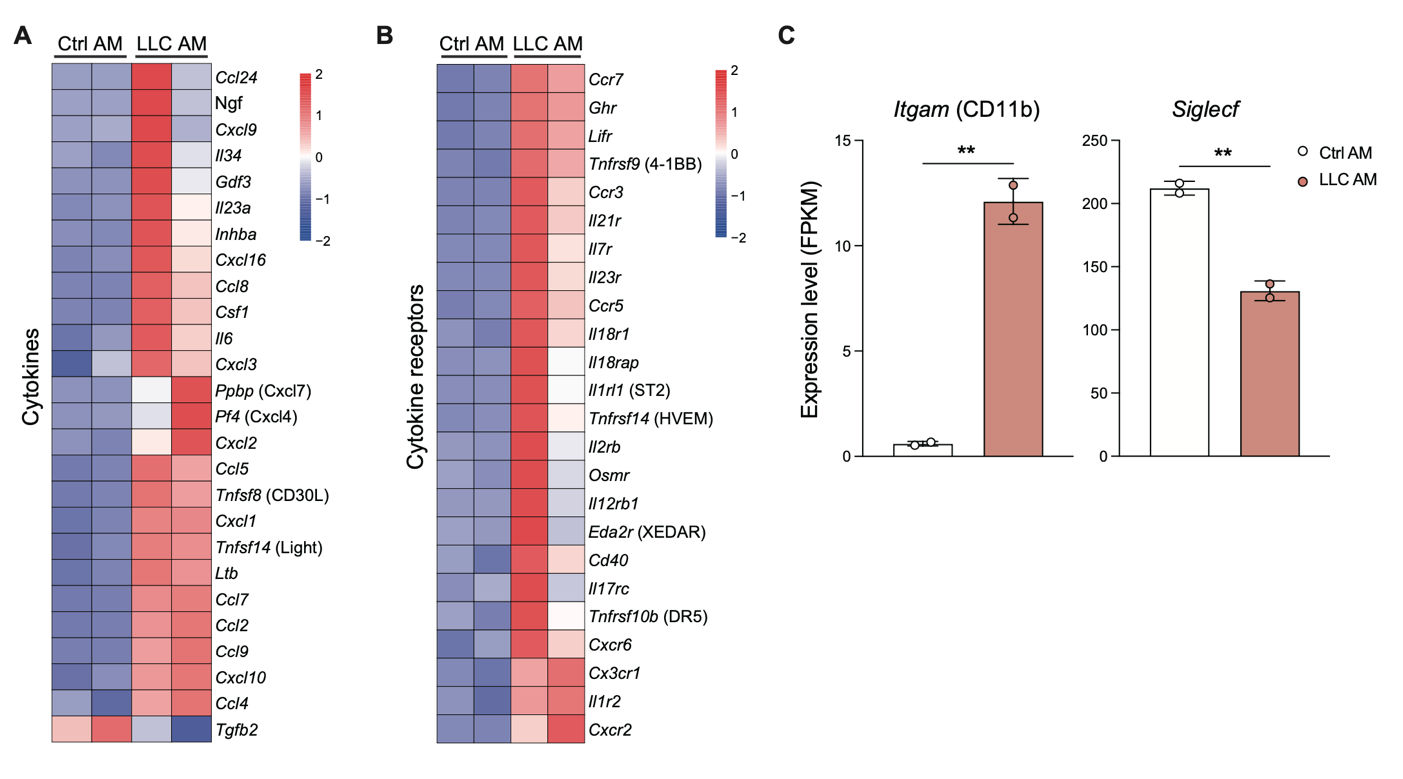


**Figure S4. Heatmap of cytokine and cytokine receptor gene-expression levels (related to Figure 5).**

(A, B) Heatmap of cytokine (A) and cytokine receptor (B) transcription levels in Ctrl AM and LLC AM. The color scale indicates the relative expression levels, with red representing higher expression and blue representing lower expression. (C) Expression levels (FPKM) of *Itgam* (CD11b) and *Siglecf* genes in Ctrl AM and LLC AM. Data are presented as means ± SD (n = 2 mice per group). Unpaired *t*-test was used, ***P* < 0.01.


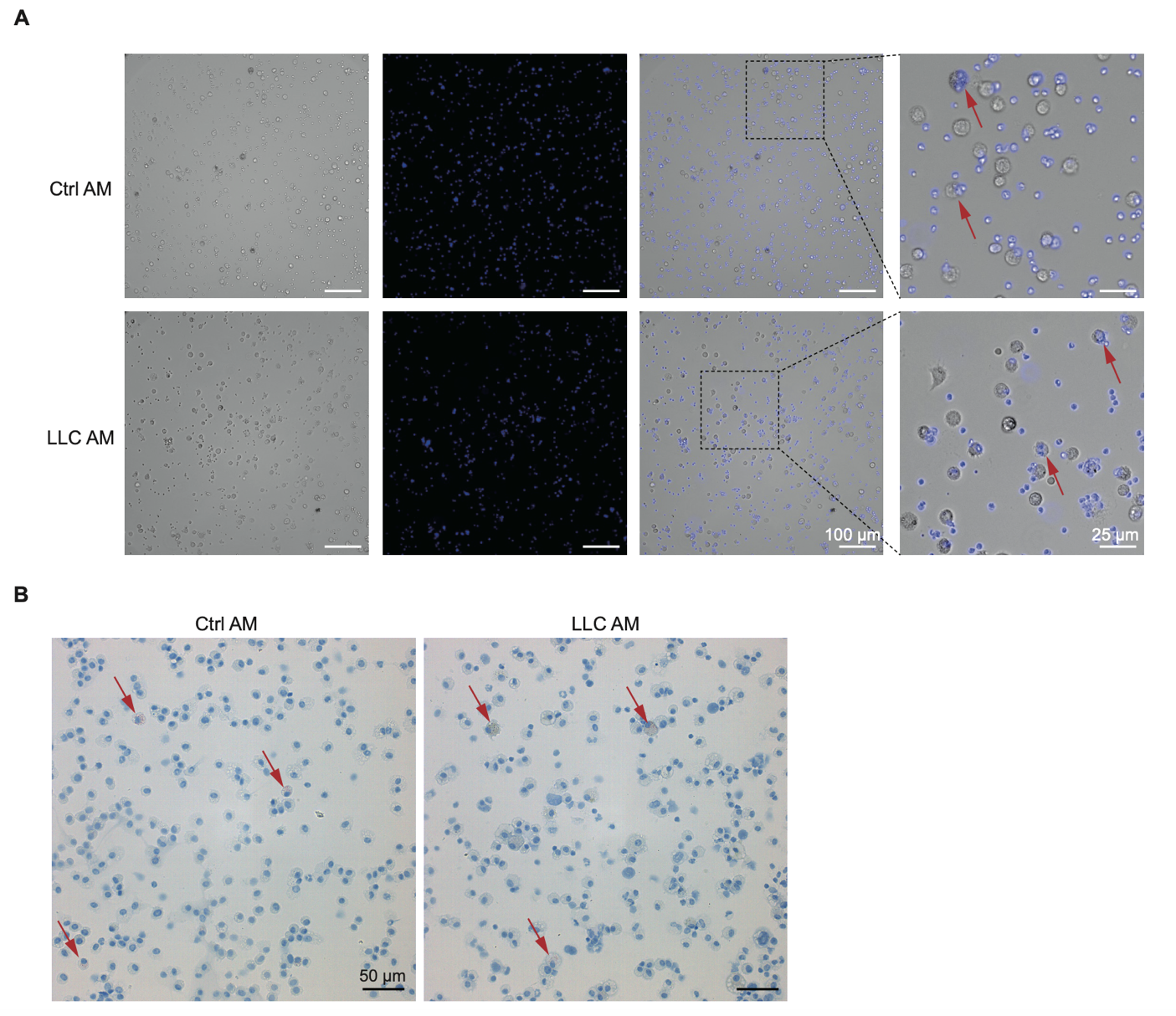


**Figure S5. Functional analysis of AMs (related to Figure 6).**

(A) Fluorescence and brightfield microscopy images of AMs from control and LLC tumor-bearing mice incubated with eFluor450-labeled apoptotic thymocytes. The dashed lines indicate the area of magnification. (B) Microscopic images of Oil Red O staining for AMs from control and tumor-bearing mice. Red arrows point to specific AMs of interest.

**
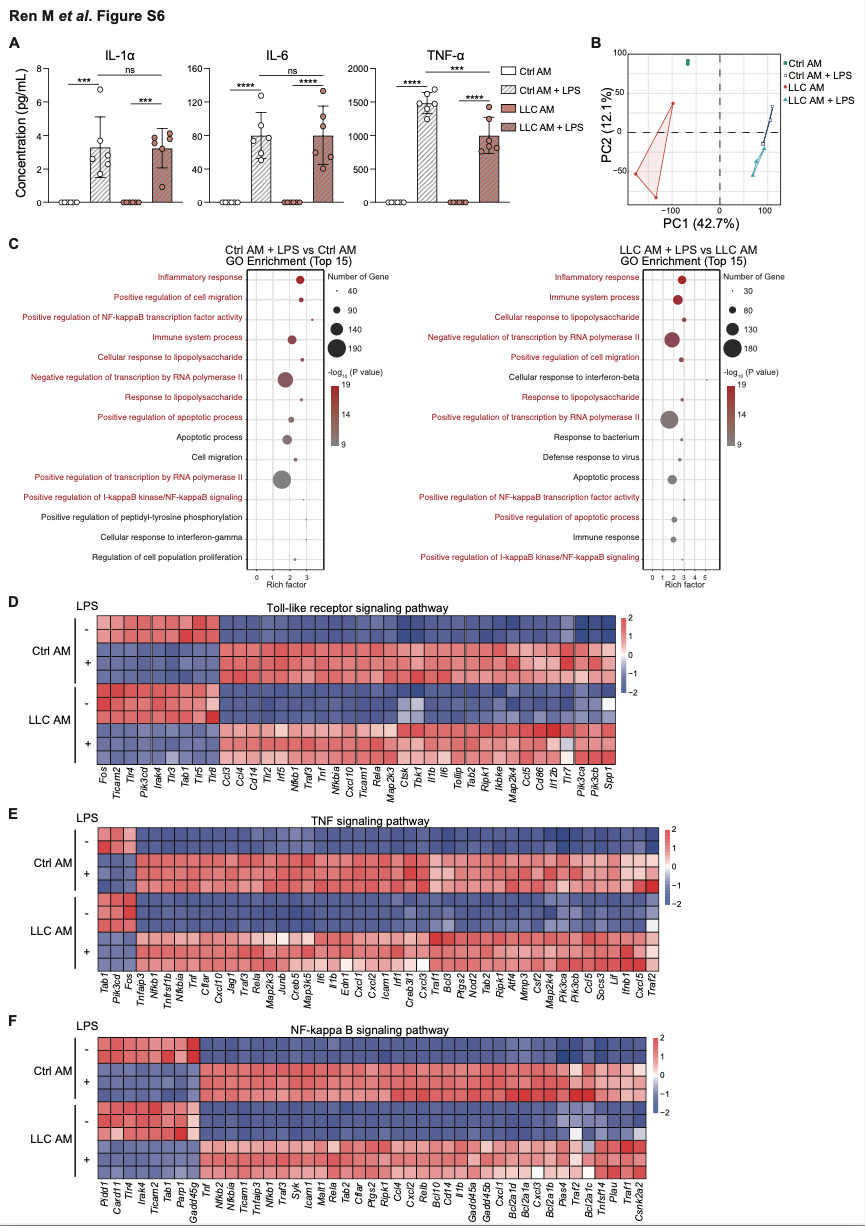
**

**Figure S6. AMs maintain the ability to respond to external stimuli in the LLC-reprogrammed ETLME (related to Figure 6).**

(A) Concentrations of IL-1α, IL-6, and TNF-α in supernatant from Ctrl AM and LLC AM treated with or without lipopolysaccharide (LPS) for 4 hours *in vitro*. (B) PCA of transcriptional profiles of the Ctrl AM and LLC AM groups with or without LPS stimulation. (C) GO enrichment analysis of the top 15 biological processes. The enrichment for DEGs in Ctrl AM (left panel) and LLC AM (right panel) upon LPS stimulation. The size of the points represents the number of genes, and the color represents the -log_10_ (P value). (D-F) Heatmap showing the expression levels of genes involved in the Toll-like receptor (D), TNF (E), and NF-kappa B (F) signaling pathways in Ctrl AM and LLC AM with or without LPS stimulation. The color scale indicates the relative expression levels, with red representing higher expression and blue representing lower expression. The data are presented as mean ± SDs in A (n = 6 samples per group). One-way ANOVA was used in A, "ns" indicates not significant, **P* < 0.05, ***P* < 0.01, ****P* < 0.001, and *****P* < 0.0001.


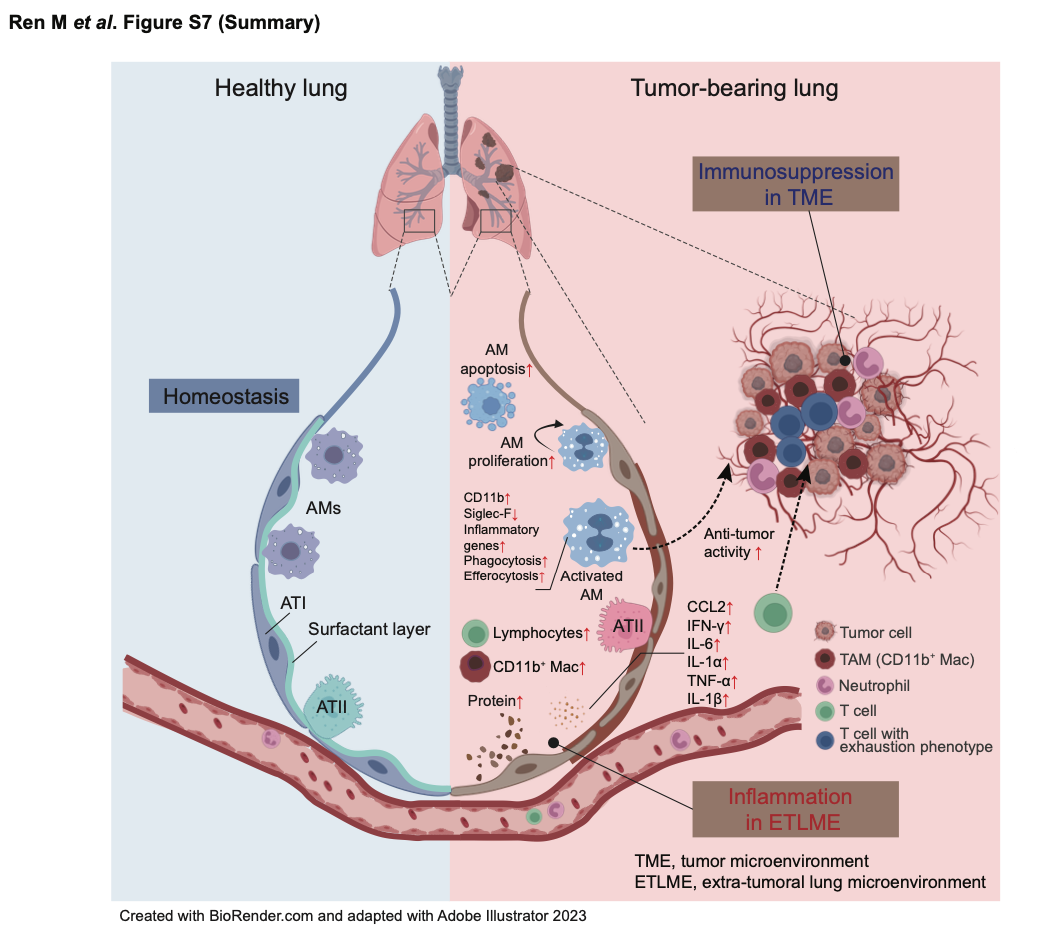


**Figure S7. AMs are activated, maintain their tissue localization, and gain enhanced anti-tumor activity in LLC-reprogrammed ETLME**

The growth of LLC disrupts the homeostatic immune microenvironment, generating two functionally and spatially different immune microenvironments in the lungs, each with a unique immune cell composition. Within TME, LLC establishes an immunosuppressive microenvironment characterized by the accumulation of CD11b^+^ tumor-associated macrophages and neutrophils, alongside a predominance of T cells with exhaustion phenotype. In contrast, the ETLME exhibits an immune-activated/inflammatory microenvironment, featuring a small amount of lymphocyte and CD11b^+^ macrophage infiltration. Here, AMs increase their proliferation capacity to counterbalance inflammation-induced cell death. Notably, AMs maintain their tissue localization without migrating into tumor lesions. In tumor-bearing lungs, AMs adopt an activated phenotype, marked by upregulation of CD11b, downregulation of Siglec-F, elevated expression of inflammatory genes, and enhanced phagocytic, efferocytotic, and anti-tumor activity.
